# CLinNET: An Interpretable and Uncertainty‐Aware Deep Learning Framework for Multi‐Modal Clinical Genomics

**DOI:** 10.1002/advs.202512842

**Published:** 2026-01-28

**Authors:** Ivan Bakhshayeshi, Mohammad Mahdi Hosseini, Ahmadreza Argha, Roxana Zahedi, Nigel H. Lovell, Hamid Alinejad‐Rokny

**Affiliations:** ^1^ UNSW BioMedical Machine Learning Lab (BML) School of Biomedical Engineering, UNSW Sydney Sydney NSW 2052 Australia; ^2^ Remote Internship in UNSW BioMedical Machine Learning Lab School of Biomedical Engineering, UNSW Sydney Sydney NSW 2052 Australia; ^3^ School of Biomedical Engineering UNSW Sydney Sydney NSW 2052 Australia; ^4^ Tyree Institute of Health Engineering (IHealthE) UNSW Sydney Sydney NSW 2052 Australia

**Keywords:** copy number variants, deep learning, gene curation, interpretability, neurocognitive disorders, uncertainty quantification

## Abstract

Identifying molecular drivers and diagnostic genes for neurocognitive disorders (NDs) remains a major challenge due to the prevalence of variants of uncertain significance (VUS) and limitations in current diagnostic platforms. While artificial intelligence (AI) offers potential solutions, existing models often lack interpretability and fail to address uncertainty, limiting clinical utility. CLinNET, a multi‐modal deep neural network with a dual‐branch design integrating sequencing data, gene expression, biological pathways, and gene ontology (GO) is introduced to enhance gene curation and VUS interpretation. CLinNET employs a biologically informed architecture, confidence‐based uncertainty quantification, and layer‐wise SHapley Additive exPlanations (SHAP) for robust interpretability. Its sparse networks, enriched with pathway and GO data, prioritize tissue‐expressed genes to improve prediction accuracy and biological relevance. Trained on ND datasets, CLinNET outperformed existing methods with an F1‐score of 76.4%, accuracy of 77.2%, and area under the precision‐recall curve (AUC‐PR) of 84%. Incorporating uncertainty filtering further improved precision to 87% while retaining 73% of predictions as high‐confidence. CLinNET identified significantly more ND‐associated genes than random permutations, with minimal overlap with cardiovascular‐associated genes, confirming specificity. Among the top decile of ranked genes, 78 were linked to NDs (*p*‐value = 1.2e‐11), and 372 to rare diseases involving nervous system abnormalities, highlighting their diagnostic potential. CLinNET's validation in prostate cancer datasets underscores its adaptability, positioning it as a robust tool for individualized medicine.

## Graphical abstract

Identifying disease‐causing genes in neurocognitive disorders remains challenging due to variants of uncertain significance. CLinNET employs dual‐branch neural networks integrating Reactome pathways and Gene Ontology terms to provide pathway‐level interpretability of genomic alterations. The framework achieves 77% accuracy (87% precision with confidence filtering), with successful validation on schizophrenia and prostate cancer. CLinNET advances precision diagnostics by linking genetic variants to actionable biological mechanisms.

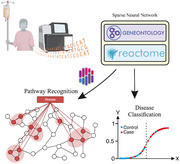

## 1 Introduction

Neurocognitive disorders (NDs), such as autism spectrum disorder (ASD) and developmental delay (DD), are marked by diverse phenotypic expressions, mainly driven by complex genetic variations.^[^ ^1^, ^2^ ^]^ These genetic alterations range from single‐gene mutations to large chromosomal rearrangements, reflecting the varied etiologies underlying NDs. As a result, individuals often exhibit impairments in cognitive, social, and motor functions, creating significant diagnostic and therapeutic challenges.^[^ ^3^ ^]^ The interplay between multifaceted phenotypes and diverse genetic mechanisms underscores the need for deeper exploration to enable early detection and targeted intervention. Numerous studies indicate that shared molecular pathways may underpin these disorders, offering valuable insights into their genetic mechanisms.^[^ ^4^, ^5^ ^]^

Recent advances in computational biology offer powerful tools to integrate and analyze multi‐modal genomic data.^[^ ^6^ ^]^ These methods have shown promise in uncovering gene–disease associations^[^ ^7^ ^]^ and revealing underlying biological processes relevant to NDs. However, many of these models struggle with interpreting variants of uncertain significance (VUS), resulting in over 50% of individuals remaining undiagnosed or receiving late diagnoses.^[^ ^8^ ^]^ Although technologies such as chromosomal microarray and whole genome sequencing have enhanced the detection of genomic variants including single nucleotide variants (SNVs) and copy number variations (CNVs),^[^ ^9^, ^10^ ^]^ a substantial subset of cases remains unresolved. Specific CNVs, including deletions at *1q21.1* and duplications at *7q11.23*,^[^ ^11^ ^]^ illustrate the complexity of identifying causative genes in regions harboring numerous candidates.

Machine learning (ML) techniques, especially deep learning, have improved CNV detection and candidate gene prioritization in NDs,^[^ ^12^, ^13^ ^]^ yet a “black‐box” effect persists, hindering clinical adoption due to limited interpretability.^[^ ^14^, ^15^, ^16^, ^17^ ^]^ Although pathway‐informed neural network (PINN) frameworks such as those developed by Elmarakeby et al.,^[^ ^18^ ^]^ Hartman et al.,^[^ ^19^ ^]^ and Prosz et al.^[^ ^20^ ^]^ demonstrate promise, they often rely on narrow knowledge sources and may overlook disorder‐ or tissue‐expressed factors essential for understanding NDs. Most importantly, these models often rely heavily on pathway analysis, limiting their capacity to pinpoint the specific genes driving pathology. Reactome^[^ ^21^ ^]^ (https://reactome.org), while extensive, covers only 56.2% of protein‐coding genes; resources like the gene ontology (GO)^[^ ^22^ ^]^ (http://geneontology.org) remain underutilized, despite offering a broader view of gene functions and interactions. Additionally, tissue‐expressed genes, known to be critical in NDs,^[^ ^23^ ^]^ are often ignored, preventing models from pinpointing which genes are most relevant in particular cellular contexts.

A further challenge lies in conveying model confidence for clinical use. Uncertainty estimation is crucial for reliable diagnostics, as overconfident predictions can lead to misdiagnosis.^[^ ^24^, ^25^ ^]^ Techniques such as Monte Carlo dropout and Bayesian neural networks help quantify uncertainty,^[^ ^26^, ^27^ ^]^ yet ensuring stable, interpretable outputs across multiple model runs remains problematic. Small variations in initialization or training can shift feature importance rankings, attribution scores, and other interpretive outputs, complicating clinical translation.^[^ ^28^, ^29^ ^]^

To address these gaps, we propose CLinNET, a novel interpretable multi‐modal model based on GO‐pathway‐informed neural network (GPINN) frameworks, designed to enhance gene curation in NDs. CLinNET integrates CNV profiles with biological pathways and GO annotations through parallel sparse networks, capturing complex genomic interactions. Tissue‐expressed approach targets the most relevant tissues and incorporates gene expression data, boosting biological validity. A layer‐wise SHapley Additive exPlanations (SHAP) structure delivers transparent, consistent interpretations, ensuring more stable insights into model decision‐making. Finally, by combining uncertainty‐aware methods with interpretable deep learning, CLinNET significantly advances ND case/control classification while elucidating the underlying genomic factors. Our approach surpasses both traditional ML and state‐of‐the‐art models, setting new standards for interpretability and clinical utility in CNV analysis, and holds promise for a deeper understanding and improved diagnosis of NDs.

## 2 Results

### 2.1 An Overview of CLinNET

CLinNET is designed to handle multiple data modalities, including SNVs, CNVs, and gene expression profiles, provided these data are available for all samples. Each modality is supplied to the model as a separate input file. For CNV data, CLinNET constructs a sample‐by‐gene matrix, assigning −1 to deletions, +1 to duplications, and 0 to unaltered regions.

**Figure 1:**
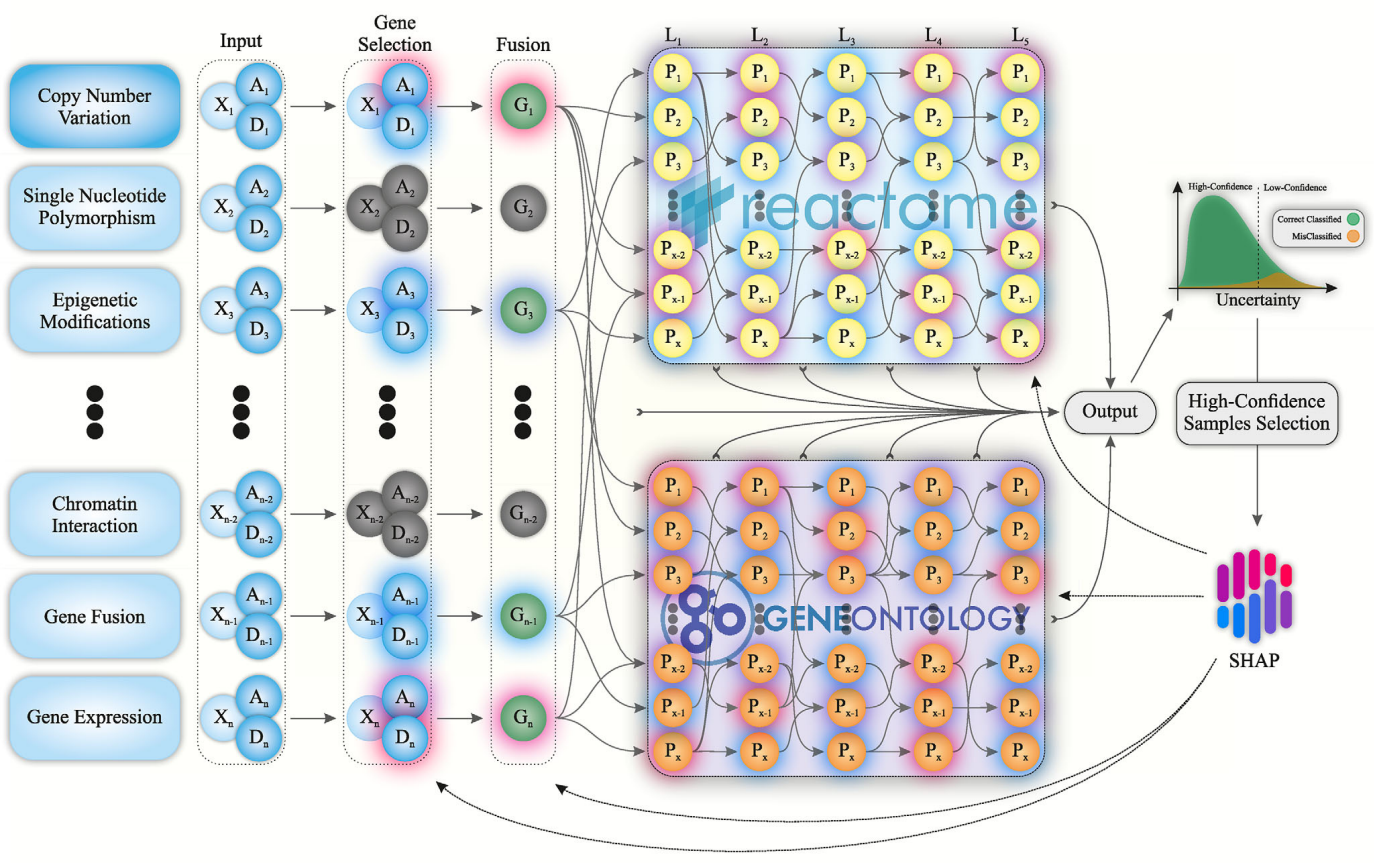
Schematic view of the CLinNET model architecture. For more details, refer to the Methods section. The model integrates multiple data sources through an input layer, including A (CNV amplification), D (CNV deletion), and X (other modalities). A dual‐network structure, informed by the Reactome and GO knowledge bases, incorporates genetic variants and biological pathway information. The G (genes) are selected through a gene selection mechanism to identify key genes associated with targeted disorders. This culminates in a binary classification output layer. Layer‐wise SHAP value extraction provides interpretable insights into model decision‐making, while confidence‐based prediction further improves model accuracy by filtering uncertain samples from the final interpretation. L1–L5 represent sparse layers within the model.

CLinNET introduces a dedicated gene selection layer (**Figure** **1**), integrating gene expression data from a curated list of genes uniquely expressed in nervous system tissues (see Experimental Section). By centering on tissues most relevant to NDs, the model's biological relevance and interpretability are enhanced. From an initial set of 18 230 protein‐coding genes, CLinNET refines its focus to 14 511 expressed specifically in nervous system tissues.

Following feature selection and data fusion, CLinNET employs a unified, multi‐modal framework built on a biologically informed architecture. CLinNET is the first GPINN model (uses parallel networks based on GO terms and Reactome pathways) to provide a comprehensive interpretation of the underlying biological mechanisms. The architecture mirrors the hierarchical structure of biological systems, processing genomic data through layers enriched by insights from 2647 Reactome pathways and 27  597 GO terms. This multi‐layered approach captures complex biological interactions, linking genetic variants to specific disease mechanisms.

To enhance both interpretability and reliability in gene curation, CLinNET incorporates a confidence‐based classification process within its biologically informed architecture. After training, the model filters out low‐confidence predictions and focuses on high‐confidence outputs. This calibrated confidence filtering improves precision, making the outputs more clinically reliable and reducing false positives. By coupling confidence‐guided sample selection with pathway‐informed modeling, CLinNET ensures that its predictions remain robust and well‐suited for clinical decision‐making.

Most importantly, CLinNET's interpretability is significantly enhanced through our layer‐wise SHAP approach, which quantifies the contribution of each feature (e.g., gene, pathway) to the model's predictions and visualizes these contributions using Sankey diagrams, where node size and color reflect feature importance. Unlike prior approaches, CLinNET is the first model to employ a comprehensive k‐fold aggregation scheme, where SHAP values are calculated across multiple model runs and then averaged. This approach addresses the inherent variability of single training runs, particularly in the case of rare disorders with heterogeneous CNVs, ensuring more consistent and reliable interpretability. This helps to achieve more stable and generalizable estimates of key features in the explainable artificial intelligence (xAI) phase.

### 2.2 Model Validation and Performance

To thoroughly evaluate CLinNET's predictive performance, we benchmarked it against well‐established ML models, including XGBoost, Random Forest, Logistic Regression, and k‐Nearest Neighbors (KNN), using ND datasets. We employed a 20‐fold cross‐validation strategy, where each fold served as the test set in turn, while the remaining folds were used for training, with one fold reserved for validation. This rigorous setup ensured minimal bias and variance across folds, providing an unbiased assessment of the model's performance. These evaluations were conducted on 47  318 samples from individuals with NDs and healthy control cases specified in the nervous system.

**Figure 2:**
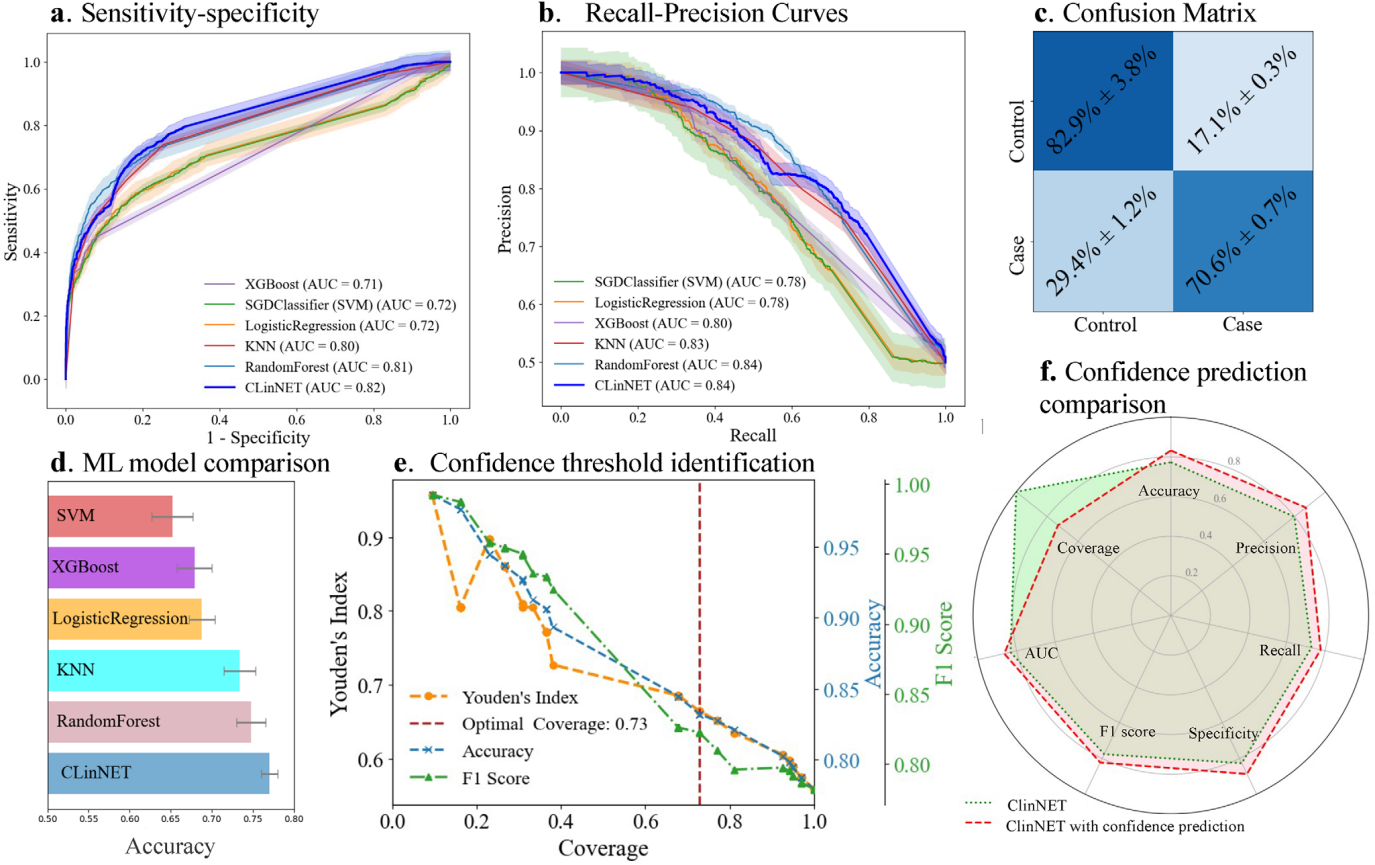
Comparative performance analysis of CLinNET against standard ML models (*n*=20 folds, 47  318 samples). a) Sensitivity–specificity curves display ROC–AUC values, highlighting CLinNET's balance with an AUC of 0.82. b) Precision–Recall curves showing AUC‐PR, with CLinNET achieving 0.84, indicating high precision across recall levels. c) Confusion Matrix for CLinNET, detailing true and false rates, illustrating accurate classification capabilities. d) Accuracy Metrics Comparison: Bar graph comparing the overall accuracy of CLinNET with other standard ML models over 20‐fold cross‐validation. This underscores CLinNET's leading performance, highlighting its practical utility in clinical settings. e) Assessment of optimal prediction confidence thresholds using Youden's Index and coverage. The figure also illustrates the variation of accuracy and F1‐score as functions of model coverage. This demonstrates the utility of a confidence‐based classification strategy to manage uncertainty in model predictions, highlighting how different thresholds can optimize model performance metrics. f) Comparison of performance metrics between CLinNET (light green) and the model with confidence prediction (pink). Key indicators highlight the enhanced performance and reliability of integrating confidence predictions.

As a result, CLinNET demonstrated superior performance across nearly all evaluation metrics, exhibiting only minimal standard deviation in its results. Notably, the model achieved an area under the precision‐recall curve (AUC‐PR) of 82% and an area under the receiver operating characteristic curve (AUC‐ROC) of 84%, as illustrated in **Figure** **2**a,b. These robust AUC values highlight CLinNET's ability to discriminate effectively between ND cases and controls, which is particularly crucial given the complexity of genomic data in these disorders. Moreover, CLinNET surpassed other models with an accuracy of 77.2%, precision of 79.1%, recall of 73.6%, and an F1 score of 76.2% (Figure 2d). Such balanced sensitivity and specificity are essential for clinical applications, where false negatives—such as overlooking a cancer or a serious ND—can have serious consequences. The model's strong F1 score underscores its effective calibration between precision and recall, reinforcing its reliability in classifying ND patients accurately.

### 2.3 Ablation Study

To assess the contributions of individual layers to CLinNET's performance and interpretability, we conducted an ablation study by systematically removing or modifying specific layers of the model. This approach enabled us to isolate the impact of each layer on the model's overall efficacy. The study was performed on a single cross‐validation fold for consistency, applying each configuration to the ND dataset, and **Table** **1** summarizes the results.

**Table 1:** Performance impact of ablation study on CLinNET.

**Model**	**Accuracy**	**Precision**	**Recall**	**F1 Score**	**AUC**
Without specific tissue	0.013	0.026	−0.008	0.008	0.003
Unrelated tissue (stroma)	−0.003	−0.005	−0.050	−0.014	−0.007
Only GO	−0.005	0.047	−0.086	−0.026	−0.004
Only Reactome pathway	−0.003	0.036	−0.065	−0.018	−0.017
Labels permuted	−0.262	−0.271	−0.265	−0.247	−0.298

We first tested the model without incorporating tissue‐expressed genes observed a slight increase in accuracy with increasing the number of input genes (+0.013%), indicating that CLinNET maintains some robustness even in the absence of this information. However, when tissue‐expressed genes were replaced with genes expressed in unrelated tissues, such as stroma, the model showed a slight reduction in accuracy (0.003%). This change highlights the importance of tissue‐expressed genes in aligning predictions with biologically relevant disease contexts despite its subtle impact on overall performance metrics.

Removing the GO or Reactome pathway layers had more pronounced effects. Omitting the GO layer reduced accuracy by ‐0.005% and recall by ‐0.086%, yet increased precision by +0.047%, illustrating a trade‐off between these metrics. Similarly, excluding the Reactome layer resulted in a ‐0.003% decline in accuracy, a ‐0.065% reduction in the recall score, and a modest +0.036% gain in precision. These highlight the significance of both GO and Reactome layers for maintaining performance and interpretability.

When label information was randomly permuted, accuracy, precision, recall, F1 score, and AUC declined by over 26%, confirming CLinNET's reliance on correct label assignments. Overall, these results underscore the critical roles of biologically informed layers and prioritizing specific tissue in ensuring high performance and clinically meaningful predictions.

### 2.4 Confidence‐Based Prediction in CLinNET

Another key innovation in CLinNET involves addressing uncertainty in its prediction, which is pivotal for reliable clinical interpretation. Misclassifications can lead to overlooked or incorrectly attributed genetic risk factors (false positives), particularly in contexts where genetic variants underpin complex diseases. To mitigate these risks, it is essential to evaluate how confident the model is in each prediction, facilitating a more cautious and informed approach to variant assessment.

By incorporating confidence‐based prediction to prioritize high‐confidence outputs, CLinNET enhances interpretability through robust SHAP‐based feature attribution. We calculate a confidence score as 2 × |*y*_*prob*_ − 0.5|, where *y*_*prob*_ denotes the predicted probability of the positive class. This score measures the distance from the decision boundary, enabling an assessment of each prediction's confidence. Predictions exceeding a predefined confidence threshold are classified as “high confidence” and retained for interpretation, while others are marked as uncertain. As shown in Figure 2e, we determine the threshold dynamically using Youden's Index and coverage metrics, balancing the trade‐off between correctly and mistakenly identified ND cases while maintaining sufficient coverage of confident predictions. Applying a 70% minimum coverage threshold identifies a subset of highly confident predictions suitable for robust SHAP analysis. This refinement increased model accuracy to 83%, with precision, recall, and F1 scores of 87%, 77%, and 82%, respectively (Figure 2f). However, coverage was reduced to 73%, leaving 27% of predictions classified as uncertain. This confidence‐based approach provides clinicians and researchers with clearer insights into the genetic and biological factors influencing CLinNET's decisions—an essential step in translating model outputs into actionable clinical outcomes where variant certainty is paramount.

### 2.5 Model Generalization Across Diverse ND Cohorts

To evaluate CLinNET's ability to generalize beyond its primary training data, we validated the model on an independent schizophrenia cohort (see Methods), comprising 443 cases and an equal number of controls. Despite the cohort's heterogeneity, CLinNET achieved an overall accuracy of 73.8%, demonstrating robust performance across distinct NDs. Notably, its precision (79.4%) and specificity (83.3%) underscored the model's effectiveness in correctly identifying control samples and minimizing false positives. Although sensitivity was moderate (64.3%), CLinNET maintained a balanced F1‐score of 71.0% and attained an AUC of 75.8%, indicating strong discriminative power.

By analyzing this unseen cohort, CLinNET identified the top candidate genes based on SHAP value including *OTUD7A*, *FOPNL*, *NIPA2*, *NIPA1*, *LRP5L*, *VWA3A*, *ZNF836*, *TUBGCP5*, and *KANSL1*. Among these, *LRP5L*, *VWA3A*, and *ZNF836* were found to be highly associated with schizophrenia. These genes are linked to key pathways involving neurotransmission, synaptic plasticity, and neuronal development, which are critical in the pathogenesis of brain‐related disorders. These findings underscore the interplay of genetic and environmental factors in schizophrenia and further demonstrate CLinNET's ability to uncover biologically meaningful insights in a diverse clinical context.

### 2.6 Benchmarking CLinNET Against a PINN‐Based Model on Prostate Cancer

We compared CLinNET, incorporating confidence‐based classification, with the state‐of‐the‐art PINN model using the prostate cancer dataset. This dataset encompasses a broad spectrum of genomic alterations, including amplifications, deletions, and single‐nucleotide mutations. The prostate cancer data was chosen because it aligns with the original development and benchmarking of P‐NET,^[^ ^18^ ^]^ providing a fair basis for comparison. Also, we synchronized random seeds across models and used class‐weighted, stratified k‐fold splits with identical folds when retraining baselines. Furthermore, this allowed us to evaluate CLinNET's ability to generalize effectively to an external dataset while addressing limitations observed with that model on our highly heterogeneous ND data.

For the prostate cancer data, we configured CLinNET to incorporate gene expression profiles specific to prostate tissue. CLinNET demonstrated modest yet meaningful improvements over P‐NET, increasing precision from 78% to 80% and accuracy from 84% to 85%. Notably, implementing CLinNET's confidence‐based prediction enhanced performance, achieving an accuracy of 93% and an F1 score of 86%. While confidence‐based prediction could improve any ML model, its integration in CLinNET serves a distinct role. Because CLinNET learns in a layer‐wise manner across genes, pathways, and biological processes, reliable intermediate activations are crucial for interpretability. Confidence gating therefore enhances not only output accuracy but also the stability of internal representations. In contrast, models trained end‐to‐end without intermediate supervision may benefit from confidence filtering only at the output level, limiting interpretability improvements. Additionally, the well‐established interpretability‐performance trade‐off^[^ ^30^ ^]^ makes CLinNET's achievement notable, maintaining competitive performance while providing enhanced biological insights.

In this comparison, while PINN‐based models rely primarily on Reactome pathways, CLinNET integrates pathway data and GO, offering broader functional insights. To address variability, CLinNET uniquely averages layer‐wise SHAP analysis results in a k‐fold aggregation scheme (see Methods), enabling a multi‐run approach that better accommodates heterogeneous CNVs. In contrast, P‐NET's non‐iterative training for producing interpretability is more prone to variability due to differences in initialization and potentially results in different top‐ranked genes.

**Figure 3:**
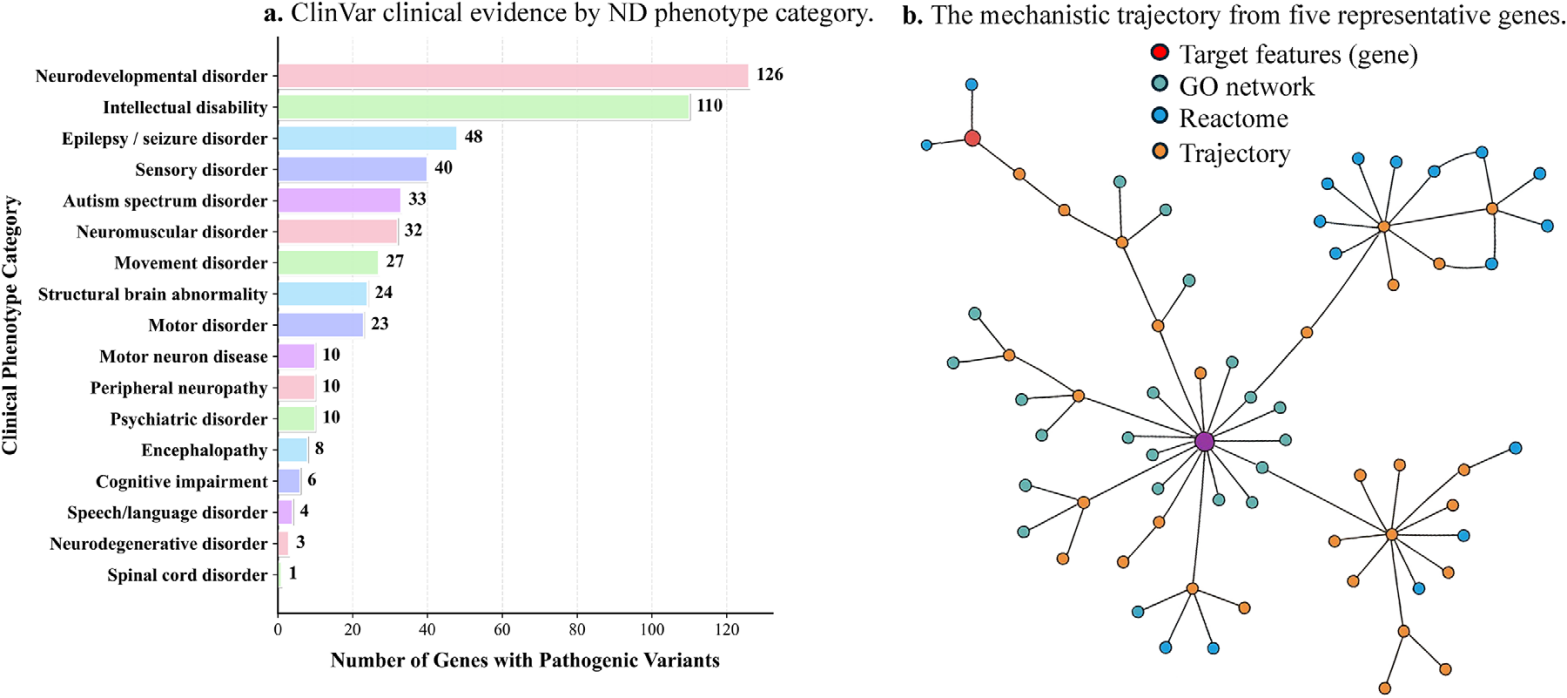
a) Clinical testing priorities in real‐world genetic diagnostics. Distribution of top 10% identified genes with confirmed pathogenic variants across 17 phenotype categories from 89 035 diagnostic cases in ClinVar. The predominance of Neurodevelopmental disorder (126 genes) and intellectual disability (110 genes) reflects actual clinical testing patterns and diagnostic priorities. b) The mechanistic trajectory from five representative genes (AR, PTEN, RB1, OBSCN, BDH2) through intermediate Gene Ontology and Reactome pathway layers to the disease phenotype node. Node colors indicate layer type (gene, pathway, or phenotype) The detailed interactive figure can be accessed via this link: https://pip‐ivan.github.io/CLinNET/interactive_network.html.

Focusing on the top 15 genes, both models identify several key genes known to play critical roles in prostate cancer progression (*AR, PTEN, TP53, RB1*), demonstrating CLinNET's ability to capture essential biological signals (Figure S4, Supporting Information). Although direct comparisons of SHAP values between feature importance are inherently complex, the consistent presence of top‐ranked genes suggests a shared emphasis on biologically relevant predictors. To further illustrate the mechanistic interpretability of ClinNET, we constructed an interactive network visualization **Figure** **3**b using the top‐ranked genes identified by SHAP analysis such as (*AR, PTEN, TP53, RB1, OBSCN, and BDH2*). This visualization traces the information flow from individual genes through Gene Ontology and Reactome pathway layers to the final disease phenotype node, providing an explicit gene–pathway–phenotype linkage. The figure highlights how alterations in these genes influence downstream pathways and ultimately contribute to disease outcome predictions.

Each model reveals distinct gene sets, underscoring potential complementary advantages. P‐NET uncovers *ANAPC16*, *APC*, *CEP70*, *CUL1*, *EIF3E*, *MAML3*, *NUP98*, *PDGFA*, *PIK3CA*, *PPP2R2A*, and *PSMD1*, many of which align with cell cycle control (e.g., *APC*, *CUL1*) and growth factor signaling (e.g., *PDGFA*, *PIK3CA*). CLinNET, however, identifies *ABCA13*, *BDH2*, *COL1A2*, *COL3A1*, *COL5A2*, *CSMD3*, *GNAS*, *IBA57*, *MUC16*, *NCAPG2*, and *OBSCN*—genes implicated in extracellular matrix remodeling, membrane transport, or intracellular signaling. For instance, *MUC16* is known for its role in immune evasion and exosome formation, potentially affecting tumor aggressiveness in prostate cancer; *COL1A2*, *COL3A1*, and *COL5A2* help shape the tumor microenvironment, which can influence metastasis; *ABCA13* encodes a large ABC transporter and may contribute to lipid raft composition relevant to exosomal trafficking.

**Figure 4:**
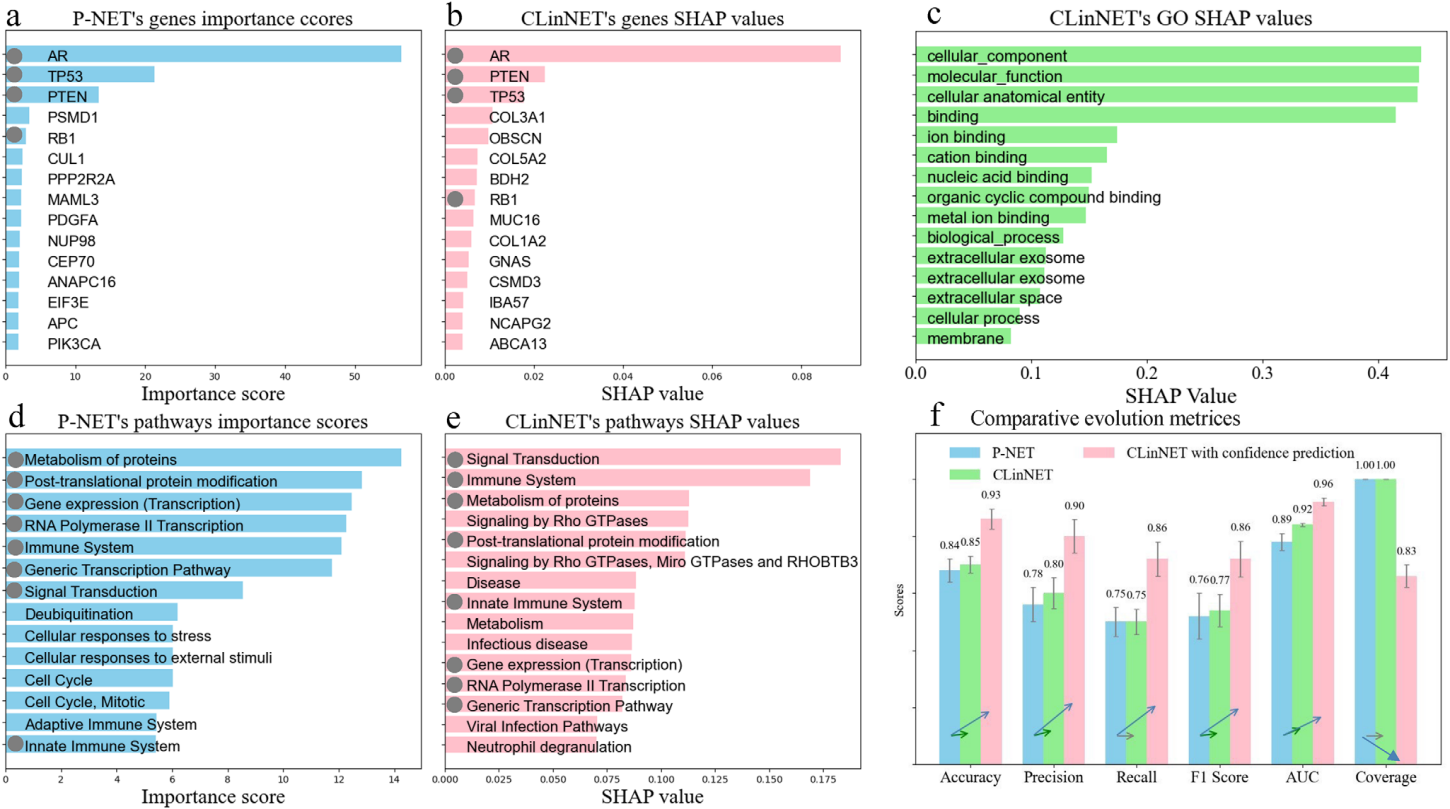
Comparative analysis of a PINN‐based model and CLinNET. a,b) Comparison of top‐ranked genes for P‐NET and CLinNET models while common genes are marked. c) The Biological process, which is provided only by CLinNET (FDR<0.05, hypergeometric test). d,e) Identified top pathways by each model. f) Comparative performance of P‐NET, CLinNET, and CLinNET with confidence prediction across key metrics and coverage on prostate cancer. CLinNET outperforms P‐NET across all evaluated metrics, while the addition of confidence prediction in CLinNET further improves performance by optimizing coverage and focusing on high‐confidence predictions (1013 prostate cancer and 47 318 ND samples).

Both models identify hallmark pathways critical to prostate cancer as shown in **Figure** **4**d,e, including “Androgen receptor signaling” and “PI3K/AKT signaling,” central to tumor initiation and progression. Shared mechanisms such as “Apoptosis” and “DNA damage response” highlight their relevance in maintaining cellular homeostasis and genomic integrity. CLinNET uniquely identifies pathways like “Signaling by Rho GTPases,” which drives cytoskeletal dynamics for tumor metastasis, and “Neutrophil degranulation,” linked to tumor progression and immune modulation within the microenvironment.

As a GBINN‐based model, CLinNET also illuminates additional GO processes central to prostate cancer biology that the PINN‐based model does not identify. For example, “Extracellular exosome” (GO:0070062) highlights intercellular communication mechanisms essential for tumor progression and metastasis, while “Metal ion binding” (GO:0046872) and “RNA binding” (GO:0003723) pertain to critical enzymatic and regulatory processes. Furthermore, “Negative regulation of epithelial cell proliferation” (GO:0050680) and “DNA‐binding transcription activator activity” (GO:0001228) underscore how CLinNET pinpoints regulatory axes that govern tumor growth and transcriptional networks. Many of the collagens (*COL1A2*, *COL3A1*, *COL5A2*) and transporter genes (*ABCA13*, *MUC16*) found exclusively by CLinNET map to these GO categories, highlighting their potential roles in suppressing malignant cell proliferation or modulating exosomal transfer of pro‐tumorigenic factors.

In parallel, we extended these experiments to the ND dataset. Although the P‐NET repository, which employs DeepLIFT for explainability, initially could not accommodate the highly heterogeneous ND data, minor package modifications only enabled us to generate comparable performance metrics. In this setting, P‐NET achieved an accuracy of 75.2%, an F1‐score of 73.1%, and recall and precision of 72.5% and 78.1%, respectively—slightly lower than CLinNET but still in alignment with the broader trends observed. These consistent outcomes illustrate the robustness and versatility of our approach across distinct disease contexts. Finally, we monitored the training progress for each individual layer in ClinNET. This not only ensures effective training but also prevents overfitting, contributing to more reliable results during the explainability stage.

Having both pathway and GO, CLinNET not only validates the principal tumor drivers identified by the P‐NET model but also uncovers additional gene sets and biological processes integral to prostate cancer progression (see Figure S4, Supporting Information). Its use of multiple SHAP iterations under an aggregation scheme ensures more stable and generalizable interpretability outcomes, addressing a key limitation of single‐run approaches. These advancements position CLinNET as a powerful tool for delivering multifaceted, biologically informed insights into disease etiology, with potential applications in clinical decision‐making and biomarker discovery.

### 2.7 Clinical Relevance of Identified Genes

**Figure 5:**
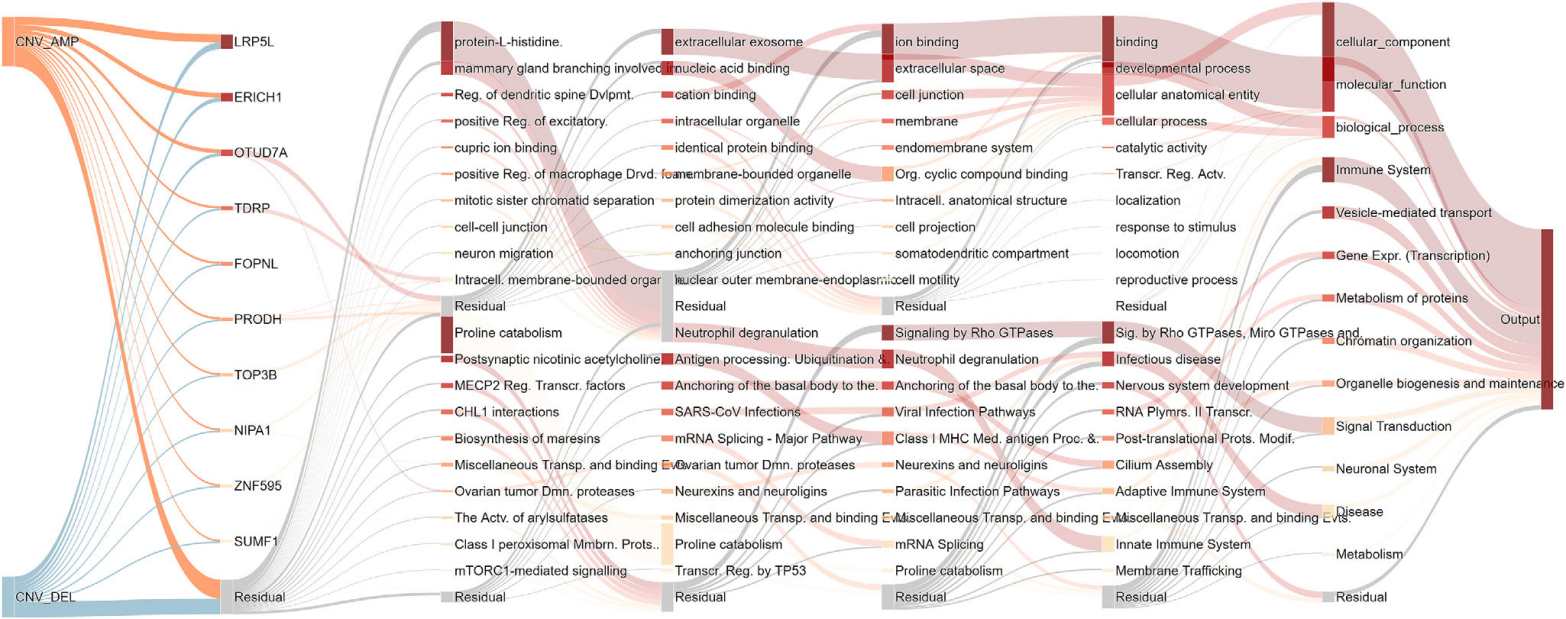
GO and pathway explainability via CLinNET's Dual‐Branch architecture. Sankey diagram illustrating pathway analysis through CLinNET's dual‐branch architecture, integrating GO and Reactome pathways. The diagram features top 10 genes ranked by aggregated SHAP values (mean across 20 folds, *n *= 47 318) (e.g., *LRP5L* and *ERICH1*), GO terms (e.g., protein‐L‐histidine and extracellular exosome), and Reactome pathways (e.g., proline catabolism and signaling by rho GTPases) in the upper and lower branches, respectively, with the remaining terms and pathways aggregated into “Residual” categories.

CLinNET's interpretability revealed several key genes with critical implications for NDs. Among the top‐ranked genes shown in **Figure** **5**, *OTUD7A*, located in the 15q13.3 microdeletion region, stands out for its association with proteasome dysfunction and schizophrenia traits.^[^ ^31^, ^32^ ^]^ Other notable genes include *FOPNL*, linked to intellectual disabilities, *PRODH*, crucial for dopaminergic regulation, and *TOP3B* and *NIPA1*, which are involved in RNA metabolism and neurodegeneration.^[^ ^33^ ^]^ Emerging candidates like *ERICH1* and *ZNF595* further illuminate the complex genetic landscape of neurocognitive regulation,^[^ ^34^ ^]^ setting the stage for a comprehensive validation of CLinNET's predictive capabilities.

To validate the clinical relevance of the genes identified by CLinNET, we conducted a comparative analysis using the Clinical Genomic Database (CGD),^[^ ^35^ ^]^ a curated repository of genetic conditions with potential medical interventions. This analysis aimed to assess the specificity of CLinNET's identified genes to NDs and their clinical utility.

**Figure 6:**
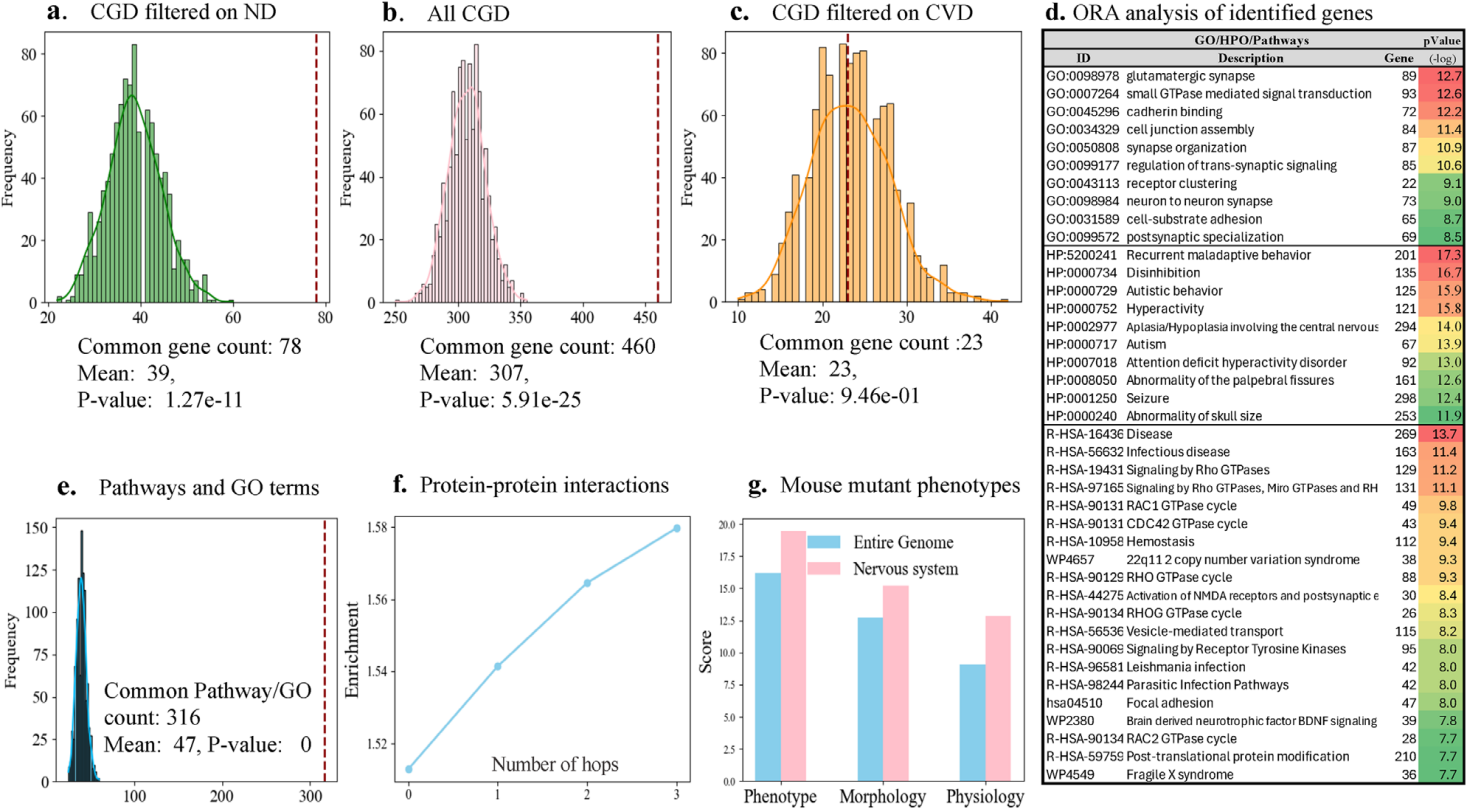
Biological characterization analysis of the top 10% of genes identified by CLinNET. a) Overlap of common genes with ND‐specific genes in the CGD permutations (*n*=1000 iterations, red line=observed). b) Intersection of common genes with the entire CGD dataset. c) Overlap of common genes with CVD‐related genes in CGD. d) ORA analysis of top genes, highlighting enriched GO terms, HPO categories, and pathways, ranked by significance (‐log10 *p*‐value) and linked to genes identified based on SHAP values (hypergeometric test, FDR<0.05, *n*=1452 genes). e) Comparison of top pathways/GO terms from CLinNET with those identified by WebGestalt over 1000 permutation iterations. f) PPI analysis showing enriched connectivity among identified genes versus random sets across various hops (0‐hop to 3‐hop). g) Analysis of mouse mutant phenotypes, demonstrating a significant association of genes with nervous system phenotypes (hypergeometric test.

We selected a subset of the top 10% of genes ranked by CLinNET's SHAP functionality, ensuring minimal redundancy in subsequent analyses. Among these genes, 78 overlapped with ND‐specific genes listed in the CGD. This overlap was significantly higher than the average number (39) of random genes overlapping with ND‐associated genes across 1000 permutations, with a *p*‐value of 1.27 × 10^−11^ (**Figure** **6**a). Additionally, 460 of the top 10% genes identified by CLinNET were associated with at least one disease in the CGD, significantly exceeding the average overlap observed in the permutations (307 genes) with a *p*‐value of 5.91 × 10^−25^ (Figure 6b). These findings indicate a robust correlation, underscoring the broad applicability and clinical relevance of CLinNET's predictions across various genetic conditions.

To further evaluate the specificity of CLinNET's gene predictions, we compared the selected subset of genes with those associated with an unrelated condition, Cardiovascular Disease (CVD), using the CGD. Only 23 genes overlapped with CVD‐associated genes, which was not significantly different from the average overlap in the permutations (mean of 23 genes) (Figure 6c). This minimal overlap and lack of statistical significance highlight the limited relevance of these genes to CVD, reinforcing the specificity of CLinNET's output to NDs.

Finally, we evaluated the pathways and GO terms identified by CLinNET. Specifically, we used the over‐representation analysis (ORA) results as validation data (details provided in the Methods section). Our analysis focused on the top‐ranked genes predicted by the model, comparing their associated pathways and GO terms with ORA results. Using the same permutation‐based approach, we quantified the overlap between CLinNET's predictions and the independent ORA findings. Notably, 316 pathways and GO terms were shared, significantly exceeding the average overlap of 48 terms observed in random permutations (*p*‐value < 0.001). This significant enrichment highlights the robustness and biological relevance of CLinNET's predictions (Figure 6e).

These analyses demonstrated that CLinNET not only effectively identifies genes with strong clinical relevance to NDs but also accurately captures key molecular pathways and generates predictions that are both clinically and biologically relevant (detailed gene overlaps are presented in the supplementary file sheets S1–S5).

### 2.8 Gene Ontology and Pathway Validation

To elucidate the biological relevance of the top 10% of genes identified by our layer‐wise SHAP from the deleted and duplicated CNVs, we conducted a comprehensive gene ORA using WebGestalt^[^ ^36^ ^]^ (http://www.webgestalt.org), as shown in Figure 6d. This analytical approach allowed us to systematically examine associations between the gene set and GO terms, human phenotype ontology (HPO) as well as pathways.

The gene ontology analysis identified several key biological processes significantly associated with NDs. The most prominent biological process, Glutamatergic Synapse, involving 89 genes with a ‐log(p) of 12.7, underscores the critical role of excitatory neurotransmission in neuronal plasticity and cognitive functions. Small GTPase‐Mediated Signal Transduction, encompassing 93 genes with a ‐log(p) of 12.6, highlights intracellular signaling processes crucial for synaptic plasticity and neural development. Cadherin Binding, with 72 genes and a ‐log(p) of 12.2, emphasizes cell–cell adhesion, essential for synaptic organization and neural network stability. Cell Junction Assembly and Synapse Organization, involving 84 and 87 genes, respectively, reinforce the importance of structural and functional integrity of synapses, while regulation of trans‐synaptic signaling with 85 genes and postsynaptic specialization with 69 genes highlight key mechanisms in maintaining neural circuitry and cognitive function. Dysregulation in these biological processes, including receptor clustering and neuron‐to‐neuron synapses, has been implicated in NDs, affecting synaptic connectivity, signal transmission, and cognitive processes.

The pathway analysis identified several biologically and clinically significant pathways associated with NDs. The top pathway, R‐HSA‐1643685, encompassing 269 genes with a ‐log(*p*) of 13.7, reflects a broad range of genetic factors implicated in ND processes. Among the other notable pathways, Signaling by Rho GTPases (R‐HSA‐194315), with 129 genes and a ‐log(*p*) of 11.2, underscores the importance of cytoskeletal dynamics and neuronal structure in ND pathogenesis. The 22q11.2 CNV Syndrome pathway (WP4657), involving 38 genes (−log (*p*) = 9.3), remains clinically significant for its link to schizophrenia and associated ND anomalies. Meanwhile, the Activation of NMDA receptors and postsynaptic events pathway (R‐HSA‐442755), covering 30 genes with a ‐log(*p*) of 8.4, highlights the critical role of excitatory neurotransmission in cognition. Notably, the brain‐derived neurotrophic factor (BDNF) signaling pathway (WP2380), encompassing 39 genes (‐log(*p*) = 7.8), is integral to synaptic plasticity and neuronal survival, while Fragile X syndrome (WP4549), with 36 genes (‐log(*p*) = 7.7), illustrates the impact of epigenetic and translational dysregulation on cognitive function. These findings demonstrate CLinNET's capability to elucidate complex molecular interactions and regulatory mechanisms underpinning NDs, offering valuable insights for targeted therapeutic strategies and improved clinical outcomes.

The HPO analysis identified several key phenotypic abnormalities significantly associated with NDs. Prominent terms include recurrent maladaptive behavior (HP:5200241, 201 genes, −log (*p)* = 17.3), disinhibition (HP:0000734, 135 genes, −log (*p)* = 16.7), and hyperactivity (HP:0000752, 121 genes, −log (*p)* = 15.8), highlighting prevalent behavioral and cognitive challenges such as Attention‐deficit/hyperactivity disorder and ASD. Autistic behavior (HP:0000729, 125 genes, −log (*p)* = 15.9) and autism (HP:0000717, 67 genes, −log (*p)* = 13.9) emphasize the social and communication deficits central to many NDs. Neurological abnormalities such as aplasia/hypoplasia involving the central nervous system (HP:0002977, 294 genes, −log (*p)* = 14.0) and seizures (HP:0001250, 298 genes, −log (*p)* = 12.4) reflect the diverse clinical presentations complicating ND diagnosis and treatment. Additionally, abnormality of the palpebral fissures (HP:0008050, 161 genes, −log (*p)* = 12.6) and abnormality of skull size (HP:0000240, 253 genes, −log (*p)* = 11.9) further illustrate the spectrum of associated phenotypic features. By mapping these phenotypes to specific genetic factors, CLinNET enhances our understanding of the molecular mechanisms driving NDs, thereby informing more precise diagnostic and therapeutic strategies.

### 2.9 Protein‐Protein Interaction Network Analysis

We conducted a protein–protein interactions (PPI) network analysis using STRINGdb^[^ ^37^ ^]^ to assess the connectivity of the top 10% of genes identified by CLinNET compared to random gene sets. As shown in Figure 6f, interestingly, 89% of the top genes were directly connected (0 hop), significantly exceeding random sets (59%; enrichment: 1.51; *p*‐value = 1.3 × 10^−10^). Connectivity increased to 92%, 93%, and 96% when including one‐hop, two‐hop, and three‐hop neighbors, respectively. This high level of interconnectedness highlights the biological relevance of CLinNET's predictions, indicating that these genes are part of coherent molecular networks critical to ND.

### 2.10 Mouse Mutant Phenotypes Analysis

Interestingly, our analysis of mouse mutant phenotypes revealed that the top 10% of genes identified by CLinNET were significantly enriched for homologs linked to nervous system‐related phenotypes in mice. For “Nervous system phenotype,” the enrichment score was 19.46 compared to a genome‐wide average of 16.18, while “Abnormal nervous system morphology” and “Abnormal nervous system physiology” showed enrichment scores of 15.24 and 12.87, respectively, exceeding baseline scores of 12.73 and 9.09 (Figure 6g). These results underscore CLinNET's ability to prioritize genes with critical functional roles in the nervous system, further supporting their relevance to NDs.

### 2.11 Real‐World Diagnostic Impact

Beyond statistical performance, clinical benefit requires demonstrable impact on diagnostic outcomes. ClinVar,^[^ ^38^, ^39^ ^]^ the largest archive of clinically‐interpreted variants (>2.5M submissions from 2000+ laboratories), provides real‐world evidence of diagnostic utility by documenting variants actually identified in patient care, their clinical actionability, and diagnostic yield across global healthcare settings. Analyzing the top 10% ClinNET ranked genes revealed 515 genes harboring pathogenic variants distributed across 17 ND categories (Figure 3a). Among these, neurodevelopmental disorders (126 genes) and intellectual disability (110 genes) represented the most prevalent categories, underscoring ClinNet's alignment with recognized neurobiological etiologies. Approximately 22% of all variants achieved multi‐laboratory consensus(Figure S5a, Supporting Information), indicating high diagnostic reproducibility across independent testing centers.

ClinNET also demonstrated substantial diagnostic efficiency (Figure S5c, Supporting Information): half of all clinically confirmed diagnoses could be captured by testing only 55% of ClinNET‐prioritized genes, representing a 45% reduction in testing burden. This efficiency directly translates to shorter diagnostic odysseys, lower costs, and improved accessibility to precision diagnostics.

Moreover, the top 20% of prioritized genes accounted for 35% of all documented clinical diagnoses, and the highest‐ranked genes corresponded to those with the greatest real‐world diagnostic frequencies (Figure S5b, Supporting Information). Notably, NF1 (8140 cases), PKD1 (2876 cases), and TSC2 (2196 cases) were among the most frequently diagnosed, each carrying the maximum clinical actionability score. This concordance confirms that ClinNET not only prioritizes genes with strong computational support but also identifies those most likely to yield actionable diagnoses in clinical practice.

## 3 Discussion

CLinNET represents a clinically oriented, multi‐modal GPINN framework that leverages a combination of Reactome and GO knowledge bases to streamline the analysis of NDs and related genetic conditions. Unlike conventional architectures that can be arbitrarily over‐parameterized, CLinNET adopts sparse, biologically constrained layers, substantially reducing trainable parameters while retaining high predictive performance. This parsimonious design not only enhances interpretability but also integrates tissue‐expressed considerations, ensuring that genes most relevant to disease are appropriately nominated based on their functional significance in the affected cellular context.

A key strength of CLinNET is its ability to handle large and heterogeneous datasets. Where comparable methods often struggle with extensive genomic variability, CLinNET adeptly processes data from multiple sources, such as CNVs, SNP, and tissue‐expressed gene profiles.^[^ ^40^ ^]^ Moreover, its application to both prostate cancer and monogenic ND cohorts illustrates the model's adaptability, accurately predicting clinically aggressive phenotypes in diverse patient populations. Such adaptability underscores the potential utility of CLinNET in uncovering convergent molecular processes that drive disease progression—a finding that could inform precision therapies in molecularly stratified groups.

CLinNET further demonstrates cross‐disorder generalizability by validating on an independent schizophrenia cohort—a distinct neuropsychiatric condition with different clinical presentation and onset relative to the training data. The model achieved strong performance despite receiving no schizophrenia‐specific training, highlighting its ability to capture shared molecular underpinnings across disorders. Moreover, its stable performance under domain shift, as shown in prostate cancer data, reflects effective knowledge transfer beyond ND contexts.

Unlike traditional pipelines that use separate statistical methods for each molecular feature, CLinNET unifies architecture weights multiple genomic determinants–mutations, CNVs, gene expression—according to their disease contributions, bolstering diagnostic precision, and biological insight. Incorporating tissue‐expressed genes further strengthens the model's plausibility by acknowledging context‐dependent patterns in ND‐associated genes. For the first time, CLinNET employs a dual‐branch design informed by both Reactome pathways and GO terms, broadening the interpretive scope beyond single‐resource constraints. This approach led to the detection of meaningful pathways relevant to NDs, including vesicle‐mediated transport, signaling by Rho GTPases, and extracellular exosome biology, which together shape crucial neurological processes. In addition, CLinNET identified clinically relevant genes that had not been previously implicated in NDs but have established roles in other genetic disorders—372 such genes in total—opening avenues for further experimental validation and potential therapeutic discovery.

A pivotal innovation in CLinNET is its layer‐wise SHAP approach, augmented by a 20‐fold aggregation scheme. This methodology addresses the variability in single‐run deep learning models by averaging feature‐importance rankings across multiple training iterations, resulting in more stable and robust interpretations. Visualizing these SHAP‐derived insights via Sankey diagrams illuminates how genetic variants and molecular processes intersect to generate disease phenotypes, effectively guiding the formulation of mechanistic hypotheses. For instance, while CLinNET rediscovers known ND‐associated genes like *PRODH*, *NIPA1*, *TOP3B*, and *OTUD7A*, it also highlights emerging candidates such as *LRP5L* and *ERICH1*. The overlap with genes in the CGD further validates their clinical relevance and reinforces CLinNET's potential for deployment in precision medicine. In clinical genomics, over‐confident models can lead to false positives that undermine patient care. CLinNET mitigates this risk through a calibrated confidence‐filtering strategy implemented through isotonic regression and threshold optimization, designed to reflect real‐world diagnostic trade‐offs. By aligning predicted probabilities with true diagnostic likelihoods, the model allows clinicians to adjust decision thresholds according to clinical context–prioritizing sensitivity when missed diagnoses carry greater consequences. This confidence‐based classification further enables clinicians to focus on high‐reliability predictions, an especially critical feature for complex or rare disorders where mis‐classification can delay intervention or affect family screening.

Furthermore, CLinNET displays strong performance across another ND cohort and adapts well to domain shifts, such as prostate cancer, indicating considerable flexibility. Nonetheless, data heterogeneity—stemming from diverse microarray platforms, variant‐calling pipelines, and clinical protocols—poses a challenge. Although CLinNET mitigates many of these issues through careful normalization and cross‐validation, more standardized genomic data generation could extend its applicability. Ultimately, ongoing assessment will be required to confirm the model's portability across a broader spectrum of disease contexts, particularly as computational methods evolve and novel data types emerge. Moreover, ClinVar validation underscores CLinNET's translational relevance, demonstrating a 45% reduction in testing burden needed to achieve comparable diagnostic yield—reflecting measurable improvements in diagnostic efficiency, accessibility, and clinical decision‐making.

Despite these advancements, CLinNET's performance depends on several key factors. As a GPINN‐based model, its reliance on Reactome and GO databases subjects the model to coverage gaps or outdated annotations, suggesting potential benefits from leveraging alternative resources like the Kyoto Encyclopedia of genes and genomes (KEGG)^[^ ^41^ ^]^ or user‐defined modules. Expanding to multi‐omics data–proteomics, metabolomics, single‐cell RNA‐sequencing—would likely enrich the model's ability to parse complex disease etiologies. While the current findings are promising, experimental validation via CRISPR‐based functional assays or in vivo models remains vital to confirm the roles of newly identified genes. In addition, prospective, multi‐site clinical studies are required to quantify decision‐level benefit (e.g., decision‐curve analysis, diagnostic yield, time‐to‐diagnosis) and to evaluate clinician uptake of interpretability outputs; until such studies are completed, CLinNET should be considered an analytical decision‐support tool rather than a diagnostic. Moreover, the model's current binary approach to tissue specificity could be refined to include single‐cell and spatiotemporal expression data, improving gene prioritization. Finally, meticulous hyperparameter tuning and the ability to replace “hardcoded” pathway structures with other relevant modules ensure that our model remains adaptable as both data and methodology evolve.

In conclusion, CLinNET exemplifies how GPINN models can revolutionize genomic analysis for complex disorders like NDs. Its capacity to integrate diverse molecular features, incorporate tissue‐specific insights, and manage uncertainty makes it a compelling framework for both research and clinical applications, paving the way for advancements in precision medicine and therapeutic development.

In conclusion, CLinNET exemplifies how GPINN models can revolutionize genomic analysis for complex disorders like NDs. Its capacity to integrate diverse molecular features, incorporate tissue‐specific insights, and manage uncertainty makes it a compelling framework for both research and clinical applications, paving the way for advancements in precision medicine and therapeutic development. Future work will focus on multi‐cohort ND validation and prospective, workflow‐level testing; until then, our calibrated, triage‐oriented outputs are intended to support—not replace—clinical judgment. All code and configuration needed for end‐to‐end reproduction are publicly available.

## 4 Experimental Section

### Data

Carefully curated CNV datasets from multiple large‐scale, high‐quality studies of ND and psychiatric disorders were utilized. The primary ND dataset was obtained from the Simons Simplex Collection (SSC)^[^ ^42^ ^]^, a key resource developed by the Simons Foundation Autism Research Initiative (SFARI) and MSSNG consortia project.^[^ ^10^ ^]^ The SSC is a well‐established and widely recognized dataset that recruited simplex ND families through 12 university‐affiliated research clinics, all of which followed standardized participant selection protocols developed by the University of Michigan Autism and Communication Disorders Center. Participant recruitment for the SSC was completed in 2011, and full cohort details, including sex distribution, ancestry, and diagnostic criteria, are publicly available through the SFARI website (https://www.sfari.org/resource/simons‐simplex‐collection) and the original publications referenced herein. Specifically, probands were required to have a clinically confirmed ND diagnosis, rigorously validated through both the Autism Diagnostic Interview‐Revised and the Autism Diagnostic Observation Schedule.^[^ ^43^ ^]^

We analyzed CNV data from a comprehensive and diverse cohort comprising 23 659 ND patients and 46421 control subjects (total of 70 080). This dataset was sourced from multiple ND‐associated CNV studies. The diversity and heterogeneity of this dataset, stemming from differences in microarray platforms and CNV‐calling algorithms, required extensive pre‐processing to ensure consistency and accuracy. The control dataset included unrelated individuals without ND diagnoses, drawn from publicly available CNV datasets. All control samples were selected based on inclusion criteria defined in the original publications, which excluded individuals with known ND or psychiatric disorders. Where available, related individuals were identified and excluded from both case and control groups to prevent potential confounding due to population structure or familial correlation.

To standardize genomic coordinates, we adopted the hg19 genome build and applied the LiftOver tool from the UCSC Genome Browser,^[^ ^44^ ^]^ followed by validation using NCBI's Remap tool. CNVs lacking precise genomic coordinates or located on the Y chromosome were excluded to preserve data integrity and ensure analytical consistency. The processed data were encoded as a sample‐by‐gene matrix using a ternary scheme: –1 for deletions, +1 for duplications, and 0 for unaltered regions. To address class imbalance, we implemented an undersampling strategy, resulting in a balanced dataset of 47 318 samples comprising both case and control groups.

To evaluate model generalizability, an independent, held‐out schizophrenia cohort of 886 samples (443 cases, each matched to one control) was used. These data were sourced from the DECIPHER database.^[^ ^45^ ^]^ Additionally, we benchmarked our model against the P‐NET model using a prostate cancer dataset from its original study. This dataset comprised 1013 samples, including 680 primary prostate cancers and 333 cases of castration‐resistant prostate cancer, each annotated with CNVs such as amplifications, deletions, and mutations.

To integrate pathway‐level information, the Reactome knowledge base was utilized, mapping the unified gene list to curated pathways. This approach provided a systems‐level context to the genetic findings. Concurrently, we incorporated GO annotations, enriching the dataset with standardized descriptors of molecular functions, cellular components, and biological processes. This multifaceted integration of CNV data with pathway and ontology information facilitated the identification of biologically relevant patterns, enhancing the interpretability of our model.

To incorporate gene expression into our model, tissue‐expressed genes were defined using a reference set derived from the integration of the FANTOM5 CAGE‐Associated Transcriptome database^[^ ^46^ ^]^ and Ensembl genome annotations (https://www.ensembl.org), both aligned to the hg19 genome assembly. The focus was exclusively on protein‐coding genes. A custom merging pipeline reconciled entries based on gene symbols and genomic coordinates, with FANTOM5 coordinates prioritized in cases of discrepancy. This yielded a unified dataset comprising 18 230 genes profiled across 347 tissues.

To identify tissue‐expressed genes, a threshold of >1 transcript per million (TPM) was applied in the relevant tissue. Genes meeting this threshold were retained for model input. To emphasize disease relevance, genes expressed in tissues implicated in the disease (e.g., gut tissues in cystic fibrosis) were assigned greater weight in the model. This tissue‐aware filtering helps focus the model on genes likely to contribute to tissue‐specific pathologies.

It was noted that this approach may include housekeeping genes with ubiquitous expression across tissues. However, the weighting mechanism based on tissue relevance reduces their influence in the final model, ensuring a stronger focus on contextually important features. Future work may incorporate tissue‐specificity indices to further refine this selection.

To assess real‐world clinical benefit, CLinNET's gene prioritization was evaluated against pathogenic variants documented in actual diagnostic cases across 2500+ clinical laboratories worldwide, as curated in ClinVar. This validation framework measured whether model predictions align with genes harboring clinically confirmed pathogenic variants, providing evidence of potential diagnostic yield and clinical actionability in practice.

### CLinNET Architecture

CLinNET employed a novel dual‐branch neural network architecture specifically designed to unravel the biological processes underlying rare and diagnostically challenging disorders, such as ND (Figure 1). This architecture (detailed in Figure S2, Supporting Information) integrated heterogeneous biological data, combining genomic variations with pathway and GO information within a hierarchical framework that reflected the inherent organization of biological systems.

The model was built upon a gene selection layer that used binary filtration to isolate genes relevant to the tissue of interest from expression datasets. Unlike traditional methods that depend on predefined expression thresholds, this approach included all genes with detectable expression in the target tissue. This inclusive strategy ensured the retention of low‐expression genes, which may play essential regulatory roles while minimizing bias caused by variations in expression levels. The resulting tissue‐specific feature map dynamically adjusts the network's focus to align with the biological context being studied. The integration of biological data occurred through a custom diagonal matrix fusion layer, followed by a sparse neural network architecture that emulates hierarchical biological processes. To mitigate the vanishing gradient problem common in deep networks, a multi‐layer output extraction method was designed and the training progress for each individual layer was monitored. By aggregating outputs from multiple layers, this approach maintained robust information flow throughout the model. The hierarchical structure of CLinNET draws from established biological knowledge bases, specifically Reactome (inspired by P‐NET) and GO. The model accepted gene matrix transposed file inputs, enabling researchers to define custom pathways and gene sets. This flexibility allowed for targeted investigation of specific biological mechanisms while maintaining the model's underlying structure. The transformation of biological data into a computational framework occurred through three primary stages:

1. Initial graph construction involved organizing Reactome and GO data into a directed acyclic graph. Nodes represented biological entities (genes, proteins), while edges denote functional relationships. This graph underwent stratification, creating layers that progress from specific molecular interactions to broader biological processes.
2. Sparse connectivity patterns between layers were implemented, where nodes connect only to immediate successors. This approach reduced computational complexity while preserving biological signal propagation pathways, thereby maintaining fidelity to underlying biological mechanisms.
3. The stratified graph translated to a TensorFlow‐based neural network, with each layer corresponding to a distinct biological level. Custom layer implementations enabled dynamic feature modulation based on biological context.

### SHAP Analysis in CLinNET

A comprehensive interpretability framework was implemented for CLinNET based on an adapted SHAP analysis that accounts for the model's hierarchical structure. The implementation extended beyond traditional SHAP applications by incorporating layer‐specific feature importance calculations within the biological hierarchy. The SHAP values were derived similarly to the distribution of payoffs in a coalition game, attributing a numerical value to each feature (e.g., genes, pathways, or biological processes) indicative of its marginal contribution toward the predictive output.

In this approach, each layer of CLinNET was treated as an independent sub‐network, allowing for the calculation of SHAP values at multiple levels of biological abstraction. Layer‐wise computation of SHAP values enabled to trace the propagation of feature importance from individual genes through to higher‐order biological processes, providing insights into the model's decision‐making process at each level of the biological hierarchy. The SHAP values at each layer were computed using its DeepExplainer method, which is specifically designed for deep neural network models with dependent features. The SHAP value for a feature *i* is calculated as:

(1) $$\phi_{i}=\sum_{S\subseteq N\setminus \{i\}}\frac{|S|!(|N|-|S|-1)!}{|N|!}\left[f(S\cup \{i\})-f(S)\right]$$

where *N* is the set of all features, *S* is a subset of features excluding *i*, and *f*(*S*) is the model output when only the features in *S* are present.

To mitigate potential bias from the over‐representation of highly connected nodes in biological networks, a novel degree‐based SHAP value normalization approach was introduced. Unlike previous methods that use raw SHAP values, the approach accounts for the inherent connectivity patterns in biological networks. The connectivity of each node was determined by summing its incoming (in‐degree) and outgoing (out‐degree) connections in the hierarchical biological networks. The normalized SHAP value is then computed as:

(2) $$\phi_{i}^{l,\text{adj}}=\frac{\phi_{i}^{l}}{d_{i}}$$

where $\phi_{i}^{l,adj}$ is the adjusted SHAP value for feature *i* at layer *l*, and *d*_*i*_ represents the total degree of node *i*. This normalization compensated for the inherent bias where highly connected nodes in the biological network might accumulate higher SHAP values simply due to their network position rather than their biological relevance to the disease mechanism. By implementing this degree‐based normalization, a more balanced interpretation of feature importance was provided that better reflected biological significance rather than network topology.

Moreover, a rigorous 20‐fold aggregation scheme strategy for SHAP value computation was employed to ensure robust feature importance estimation. This strategy helped account for data variability, providing more stable and generalizable SHAP value estimates.^[^ ^47^ ^]^ For each fold, wSHAP values were calculated independently and then their arithmetic mean across all folds was computed:

(3) $$\phi_{i}^{\text{comb}}=\frac{1}{K}\sum_{k=1}^{K}\phi_{i}^{k}$$

where $\phi_{i}^{\text{comb}}$ represents the combined SHAP value for feature *i*, *K* is the total number of folds (*K* = 20), and $\phi_{i}^{k}$ denotes the SHAP value from fold *k*. This straightforward averaging approach ensured stable and representative feature importance estimates while accounting for variations across different data partitions.

A SHAP‐based graph was constructed to visualize the propagation of SHAP values across CLinNET's layers, offering insights into the model's internal dynamics and feature influence on predictions. To simplify these complex interactions, a Sankey diagram was designed where the flow width reflects SHAP value magnitudes, highlighting how feature relevance evolves through the network. This approach provided a clear understanding of critical pathways linking genetic features to biological processes, enabling a deeper interpretation of the genetic architecture underlying the targeted disorder. Complementing this, an interactive network visualization module was developed that enables dynamic exploration of gene‐pathway‐phenotype trajectories. This tool allowed researchers to trace complete information flow from individual genes through intermediate GO and Reactome pathway layers to the final disease phenotype prediction, with color‐coded nodes distinguishing target genes, intermediate pathway nodes, and the root phenotype node. The system supported rank‐based filtering using SHAP importance values, neighborhood exploration around genes of interest, and interactive node manipulation including click‐to‐expand functionality and dynamic floating node removal, providing mechanistic insights into how genetic variants propagate through biological pathway systems to influence disease outcomes.

### Uncertainty Mitigation

For effective uncertainty mitigation in the model, we first calibrate the predictions and subsequently identify an optimal cutoff point to handle uncertain predictions. This initial step ensures accurate and reliable probabilistic outputs, laying a strong foundation for uncertainty reduction.

To achieve this, isotonic regression (IR) was employed, a non‐parametric calibration method that avoids assumptions about the underlying data distribution. IR constructed a piecewise constant function that aligned model predictions with observed outcomes, making it particularly effective for capturing non‐linear relationships between uncalibrated probabilities and actual outcomes. Compared to parametric methods like Platt Scaling, IR provided greater flexibility and accuracy in calibration.^[^ ^48^, ^49^ ^]^ The IR process minimized the following objective function:

(4) $$\sum_{i=1}^{n}{\left(y_{i}-{\hat{y}}_{i}\right)}^{2}\;\text{subject to}\;{\hat{y}}_{i}\leq {\hat{y}}_{j}\;\text{for all}\;i<j$$

where *y*_*i*_ represents the observed outcomes, ${\hat{y}}_{i}$ are the calibrated predictions constrained to be monotonically non‐decreasing, and *i* and *j* denote the indices of observations. This formulation ensures that the fitted values adhere to the monotonicity constraints required by isotonic regression.

The calibration process began by extracting predicted probabilities from CLinNET and fitting them to the observed outcomes using IR. This calibrated model, trained on a validation set, was subsequently applied to both training and validation datasets, ensuring consistency and reliability across different subsets. Such robust calibration was critical in precision medicine, where the accuracy of probabilistic predictions directly impacted clinical decision‐making.

Following calibration, the model's interpretability and uncertainty mitigation were refined by identifying an optimal prediction cutoff point. This was achieved through confidence ranking of the probabilistic outputs, generated using the Sigmoid activation function $\psi \left(z\right)=\frac{1}{1+e^{-z}}$, which mapped the model's raw output to probabilities (*y*_prob_). To distinguish between positive (case) and negative (control) classes, the optimal threshold (th*) was determined by maximizing classification accuracy, as defined in the following function:

(5) $$th^{*}=\arg\max_{th}\;\text{Accuracy}\left(y_{\text{true}},y_{\text{pred}}\left(th\right)\right)$$

where *y*_pred_(*th*) = 1 if ψ(*z*) > *th* and 0 otherwise, with *y*_true_ representing the true labels and ψ(*z*) being probabilistic predictions (*y*_prob_). This approach is crucial for ensuring a balance between precision and recall, essential attributes for binary classification.

To manage prediction uncertainty, a confidence‐based classification strategy was implemented, incorporating an uncertainty filtration mechanism. By retaining only the most reliable predictions in the xAI phase by SHAP, CLinNET achieved a higher degree of confidence in interpretability, aligning the final predictions with biological plausibility and enhancing the model's ability to discern genuine biomarkers from noise. The selection of an optimal cutoff point incorporated a composite score combining the Youden index (*J* = Sensitivity + Specificity − 1) with model coverage, which reflected the proportion of data for which predictions are made:

(6) $$S=w_{J}\cdot N\left(J\right)+w_{C}\cdot N\left(C\right).$$

Here, *w*_*J*_ and *w*_*C*_ are weights assigned to Youden's index and coverage, respectively, while *N*(*J*) and *N*(*C*) represent their normalized values. The goal is to identify a cutoff point that optimizes this score, striking a balance between diagnostic accuracy and coverage. The employment of the KneeLocator tool enabled dynamic threshold optimization by analyzing the inflection point on Youden's index curve.^[^ ^50^ ^]^ Through this process, the model precisely targeted the trade‐off between sensitivity and specificity within a predefined coverage range, enhancing diagnostic accuracy while maintaining robust prediction confidence across the dataset. Moreover, the calibration framework mirrors real‐world diagnostic decision‐making, allowing explicit adjustment of thresholds to minimize false negatives in clinically sensitive contexts. Such refinement strengthens CLinNET's ability to discriminate outcomes accurately and underscores the importance of technical precision in advancing precision‐medicine applications.

### Training and Evaluation

CLinNET, built on robust ML techniques within the TensorFlow and Keras frameworks,^[^ ^51^ ^]^ was specifically designed to unravel the complex interplay between genetic factors and tissue characteristics. The model was trained using the Adam optimizer with an initial learning rate of 0.001. To accelerate convergence, dynamic learning rate adjustment was implemented via defined callbacks. The training process spanned 200 epochs, with the same number for the batch size, balancing thorough data exposure with computational efficiency. Dropout rates were carefully varied (0.05 to 0.1) to prevent over‐fitting. For robust validation of the model's performance and generalization, a k‐fold cross‐validation method was utilized, dividing the dataset into 20 subsets to ensure each fold maintained a balanced mix of disease and healthy samples during training and evaluation. Performance was rigorously quantified using key metrics like the F1 score, accuracy, AUC, recall, and precision, calculated as follows:

(7) $$\begin{array}{cc} \text{Accuracy} & =\frac{\text{TP}+\text{TN}}{\text{TP}+\text{FP}+\text{TN}+\text{FN}},\;\text{Precision}=\frac{\text{TP}}{\text{TP}+\text{FP}}\\ \text{Recall} & =\frac{\text{TP}}{\text{TP}+\text{FN}},\;\text{F1}=2\times \frac{\text{Precision}\times \text{Recall}}{\text{Precision}+\text{Recall}}. \end{array}$$

where true positive (TP) denotes correctly identified ND cases, true negative (TN) represents accurately classified controls, false positive (FP) indicates controls misclassified as ND, and false negative (FN) represents missed ND cases.

To assess the generalizability and adaptability of CLinNET, we applied the model to different datasets beyond the initial training scope. We conducted extensive cross‐validation on the ND dataset to confirm the model's effectiveness across the dataset. Following this, we tested the trained model on an entirely unseen cohort of schizophrenia cases to further validate its applicability to varied clinical scenarios. Additionally, we benchmarked CLinNET on prostate cancer data using a domain adaptation strategy that involved retraining the model on prostate datasets. This comprehensive approach allowed to not only test CLinNET's direct application to new datasets but also its capacity to adapt through targeted training, thereby enhancing its utility across diverse medical domains.

All procedures were executed on a high‐performance computing system equipped with 512GB RAM and an NVIDIA RTX 6600 with 48GB GPU, handling the extensive demands of training intricate models on large genetic datasets. For complete transparency and reproducibility, detailed training logs, learning curves, and performance metrics are documented in the supplementary materials (Figure S3, Supporting Information) and made available on our GitHub repository.

### CLinNET Interpretation Validation

#### Monte Carlo Permutation‐Based

A Monte Carlo permutation‐based statistical framework to validate the biological relevance of the top‐ranked features identified by CLinNET. This method evaluated the non‐randomness of gene set overlaps by comparing the observed overlap of high‐SHAP‐value features with a null distribution derived from random permutations. Specifically, the top 10% of features ranked by SHAP values were focused, representing genes, pathways, and GO terms most relevant to the model's predictions. For the primary analysis, the overlap between these top‐ranked features and neurological disease‐associated genes from the CGD was calculated as the observed value (*X*_observed_). To generate the null distribution, we conducted *N* = 1000 iterations, each time randomly sampling feature sets of the same size as the top 10% from the non‐overlapping gene pool. For each permutation *j*, the overlap with CGD entries was computed, forming the null distribution: $\left\{X_{\text{random}}^{1},X_{\text{random}}^{2},\cdots ,X_{\text{random}}^{N}\right\}$.

The statistical significance of the observed overlap was assessed using a *z*‐score and a *p*‐value, calculated as:

(8) $$Z=\frac{X_{\text{observed}}-\mu }{\sigma },\;p=\frac{\text{count}(X_{\text{random}}\geq X_{\text{observed}})}{N}$$

where μ and σ represent the mean and standard deviation of the null distribution, respectively, and *N* is the total number of permutations. The *p*‐value indicates the proportion of permutations where the random overlap exceeded or equaled the observed overlap. This framework provided robust statistical validation of the biological relevance of high‐SHAP‐value features by identifying significant overlaps with disease‐associated genes and pathways. Additionally, it highlighted common genes and pathways enriched in the top‐ranked features, ensuring their relevance to NDs.

To establish specificity for neurological conditions, similar analyses were performed using CVD genes and the complete CGD gene set as controls. This comparative approach enabled discrimination between neurological‐specific associations and general disease gene enrichment patterns. The control analyses followed identical permutation protocols, maintaining consistent sample sizes and iteration counts.

To further assess the biological significance of the GO terms and pathways uncovered by the model, the permutation framework was extended into deeper network layers. This secondary analysis utilized reference datasets derived from the enrichment analysis by the WebGestalt tool. By comparing our findings with these external enrichment profiles, an orthogonal validation method was incorporated that independently confirmed the pathways and GO terms suggested by our model.

Each permutation iteration in the pathway and GO term analysis maintained the same rigorous statistical criteria applied in the gene‐level analysis. For each test, the significance of overlaps between identified genes and reference pathways was evaluated alongside the aggregate SHAP scores of the overlapping genes in the top decile. These complementary metrics–overlap significance and SHAP‐based relevance—provided a robust framework for highlighting biological importance. To ensure a comprehensive assessment, the significance of these findings was benchmarked against null distributions generated from frequency‐based randomizations.

#### Over‐Representation Analysis

The WebGestalt platform was to conduct ORA on the top 10% of genes (all protein‐coding) ranked by SHAP values, representing the top 1452 features based on SHAP values. This threshold balanced biological relevance with statistical robustness, avoiding overly large gene sets that may dilute pathway associations while capturing critical biological processes.

The ORA utilized databases including GO categories (Biological processes, cellular components, and molecular functions), KEGG, Reactome, Panther, WikiPathways, and HPO. Enrichment was assessed using a hypergeometric test with Benjamini–Hochberg false discovery rate (FDR) correction, applying an FDR threshold of <0.5. The background set comprised all protein‐coding genes in the human genome. Enrichment terms were further filtered based on *p*‐value >0.05, sorted from low to high, and visualized using −log_10_(*p*) values to highlight significance. A complementary ORA was conducted using the gene set enrichment analysis (GSEA)^[^ ^52^ ^]^ platform to validate these findings, focusing on pathways and GO terms related to NDs. The agreement between WebGestalt and GSEA results strengthened the reliability of the analysis, confirming that the identified pathways and GO terms were not biased.

#### Protein–Protein Interaction Network Analysis

To investigate the molecular interactions of the top 10% of candidate genes identified by CLinNET, a comprehensive PPI analysis was performed using the STRINGdb. First, direct (0‐hop) interactions for each candidate gene were mapped, restricting the initial network to high‐confidence, experimentally validated connections. The network was then incrementally expanded by 1‐hop, 2‐hop, and 3‐hop neighbors, capturing broader connectivity patterns beyond immediate interactions. This iterative expansion allowed to quantify each candidate gene's embedding within the higher‐order molecular network.

#### Mouse Mutant Phenotypes Analysis

The phenotypic relevance of candidate genes was investigated using mouse model data curated from the mouse genome informatics (MGI) database (http://www.informatics.jax.org). Genes linked to the nervous system were extracted from three key phenotype categories: Nervous system phenotype (MP:0003631), Abnormal nervous system morphology (MP:0003632), and Abnormal nervous system physiology (MP:0003633). For each category, the corresponding HTML pages were systematically parsed to retrieve gene lists. A comparative analysis revealed the enrichment of CLinNET genes in these categories compared to the entire genome. Mouse‐to‐human ortholog mappings were incorporated using MGI's HGNC homologs report, allowing us to map murine findings to their human counterparts. This analysis provided crucial insights into the functional roles of ClinNET candidate genes in nervous system‐related disorders, supporting their prioritization for further translational research.

### Clinical Benefit Assessment and Real‐World Validation

CLinNET's top 10% prioritized genes (*n* = 515) were analyzed against 89 035 pathogenic variants from ClinVar (accessed November 2025) representing real‐world diagnostic evidence from 2500+ clinical laboratories. Variants were classified using ClinVar's standardized clinical significance categories (pathogenic, likely pathogenic, benign/likely benign, VUS) and assigned evidence quality scores based on review status: practice guideline (4), expert panel (3), multiple submitters (2), single submitter (1), or no assertion (0).

For each gene *g* with rank *r*, pathogenic variant count *V*_path_(*g*), maximum evidence score *E*_max_(*g*), and neurodevelopment‐specific variant count *V*_ND_(*g*) were computed across 17 clinical phenotype categories (intellectual disability, epilepsy, autism spectrum disorder, movement disorders, structural brain abnormalities, etc.). Diagnostic classification was defined as:

(9) $$D\left(g\right)=\left\{\begin{array}{cc} 1 & \text{if}\;V_{\text{path}}\left(g\right)>0\wedge E_{\max}\left(g\right)\geq 2\\ 0 & \text{otherwise} \end{array}\right.$$

Clinical utility was quantified through three metrics:

(10) $$\text{Diagnostic Yield}=\frac{\sum_{g=1}^{N}D\left(g\right)}{N}\times 100\%$$

(11) $$\text{Phenotype Specificity}=\frac{\sum_{g=1}^{N}V_{\text{ND}}\left(g\right)}{\sum_{g=1}^{N}V_{\text{path}}\left(g\right)}\times 100\%$$

where *N* is the total number of prioritized genes. Statistical validation employed Spearman's rank correlation (ρ) between gene rankings and clinical evidence burden, with significance assessed at *p* < 0.05. Clinical evidence profiles were visualized through integrated heatmaps displaying pathogenic variant counts, evidence scores, and phenotype‐specific frequencies for top‐ranked genes.

### Statistical Analysis

Data preprocessing procedures are detailed in the Data and Clinical benefit assessment subsections above. All continuous variables were presented as mean ± standard deviation (SD), with categorical data reported as counts and percentages. Primary sample sizes were: ND dataset (*n* = 47318 balanced samples), schizophrenia validation (*n* = 886), prostate cancer benchmark (*n* = 1013), and ClinVar validation (*n* = 89 035 pathogenic variants across 1452 top‐ranked genes).

Model performance comparisons employed 20‐fold stratified cross‐validation with paired *t*‐tests for metric differences and Bonferroni correction for multiple comparisons (α = 0.05/*k*, where *k* = number of comparisons). Ablation studies used single‐fold evaluation with percentage changes from baseline. Confidence intervals (95% CI) were calculated using bootstrap resampling (*n* = 1000 iterations) where indicated.

Gene set enrichment significance was evaluated through Monte Carlo permutation testing (*n* = 1000 iterations), calculating empirical *p*‐values as the proportion of random overlaps exceeding observed values. Pathway overrepresentation utilized hypergeometric testing with Benjamini–Hochberg FDR correction (FDR<0.05). PPI enrichment employed STRINGdb v11.5 with experimentally validated edges (confidence score > 0.7).

Clinical validation metrics included Spearman's rank correlation between CLinNET gene rankings and ClinVar evidence burden (ρ, *p* < 0.05), while diagnostic yield and phenotype specificity were calculated as described above. A multi‐submitter consensus (evidence score ⩾2) was used as the threshold for clinical actionability assessment.

All analyses were performed using Python 3.9.21 with SciPy 1.13.1 (permutation tests, *t*‐tests), scikit‐learn v1.6.0 (cross‐validation, isotonic regression), and custom scripts for ClinVar integration. The complete computational environment is specified at https://github.com/pip‐Ivan/CLinNET/blob/main/environment.yml.

## Conflict of Interest

The authors declare no conflict of interest.

## Author Contributions

I.B. and M.M.H. contributed equally to this work. H.A.R. conceptualized the project idea. I.B. drafted the manuscript. I.B. and M.M.H. conducted all the experiment(s), prepared the figures and tables, and analyzed the data. H.A.R., A.A., M.M.H., R.Z., and N.H.L. reviewed and edited the manuscript. All authors discussed the results and provided critical feedback on the paper.

## Supporting information



Supporting Information

## Data Availability

The data that support the findings of this study are available on request from the corresponding author. The data are not publicly available due to privacy or ethical restrictions.
