# Supplementary material for: CLinNET: An Interpretable and Uncertainty‐Aware Deep Learning Framework for Multi‐Modal Clinical Genomics
Source: Adv Sci (Weinh). 2026 Jan 28;13(12):e12842. doi: 10.1002/advs.202512842 (PMC12948272; doi:10.1002/advs.202512842)
Supplement: Supplementary file 1 — Supporting Information [file ADVS-13-e12842-s001.pdf]

8 Supplementary materials

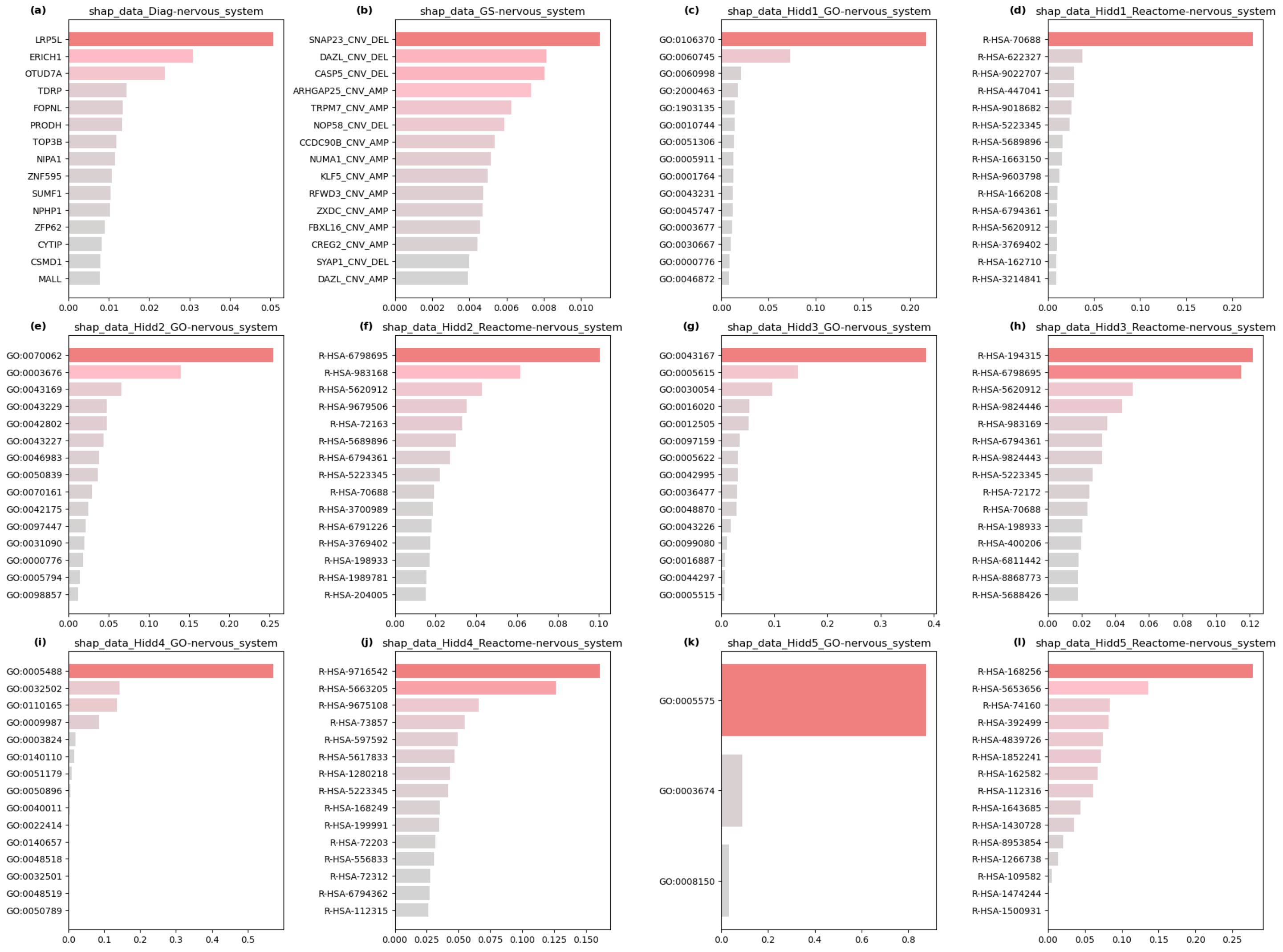

Figure S1: a to l: Figures display genes, GO terms, and pathways ranked by their SHAP values for each layer, highlighting the contribution of key features to the model's interpretability and predictive insights.

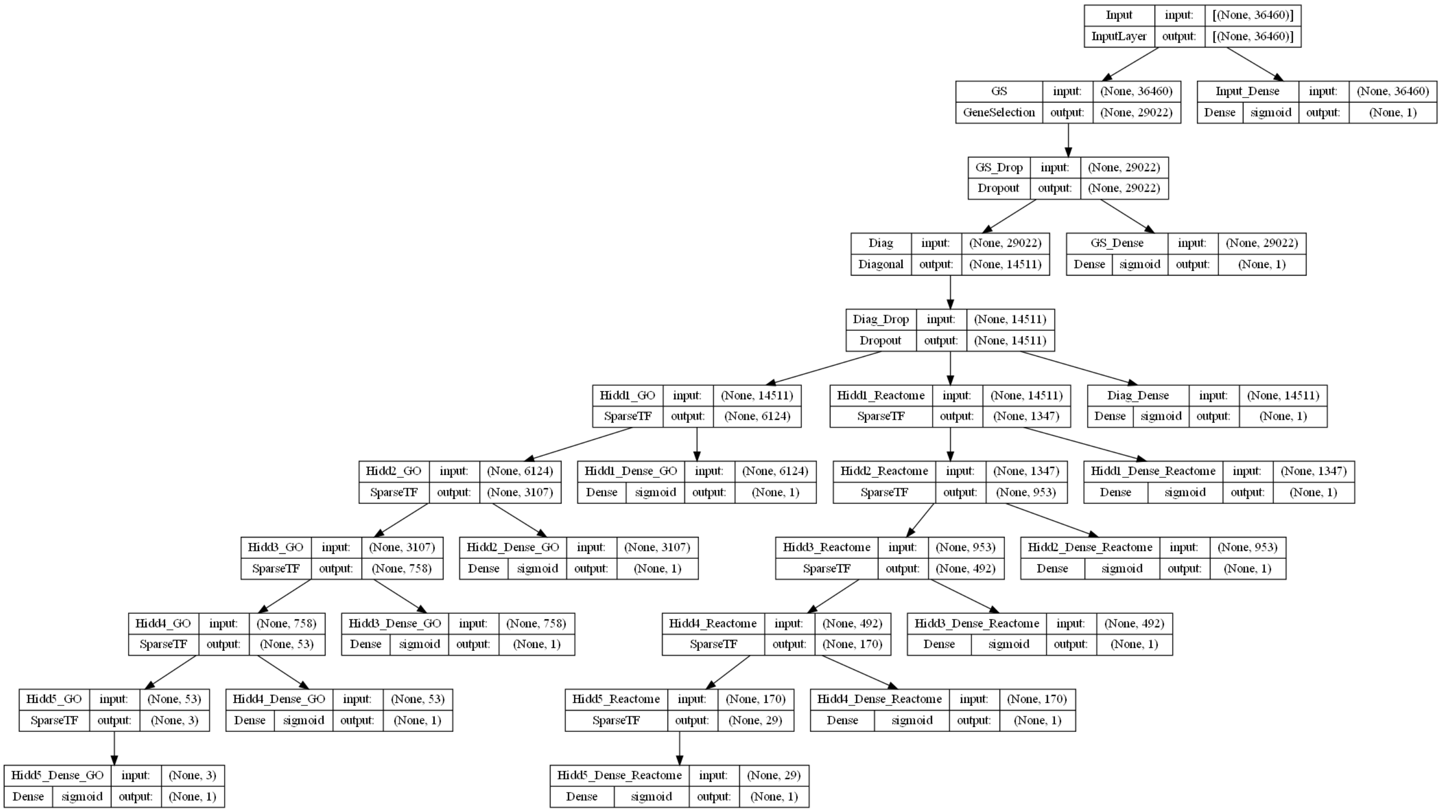

Figure S2: The figures illustrate the architecture of the ClinNET model, featuring two branches of bio-informed layers guided by GO and Reactome pathways, designed to enhance biological interpretability and predictive performance.

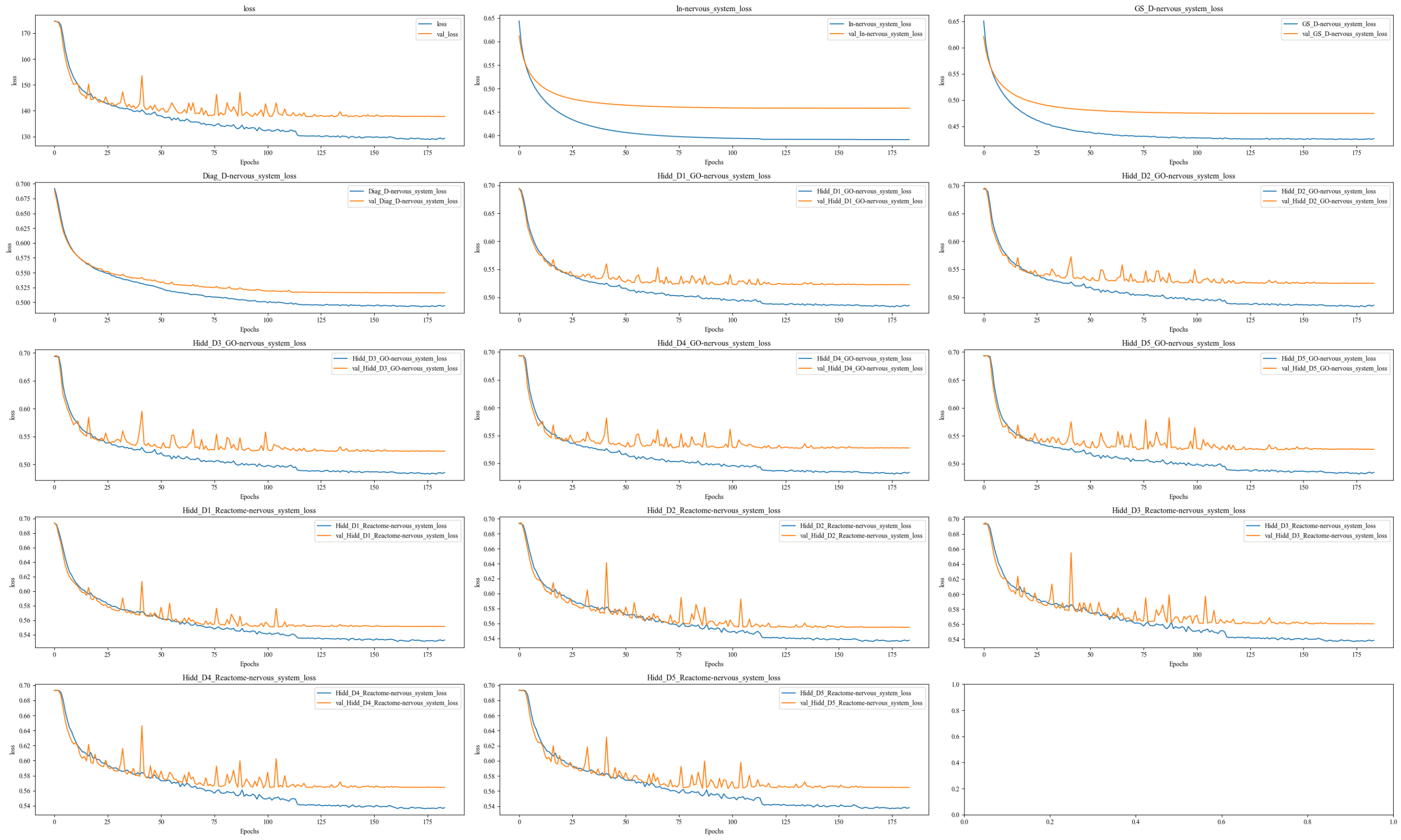

Figure S3: **a** to **l**: The figures depict the loss history for each layer of ClinNET during training. The results demonstrate that all layers were trained effectively, with no signs of overfitting, indicating balanced learning across the network.

a. ClinNET interpretation covers both pathways and GO for prostate cancer.

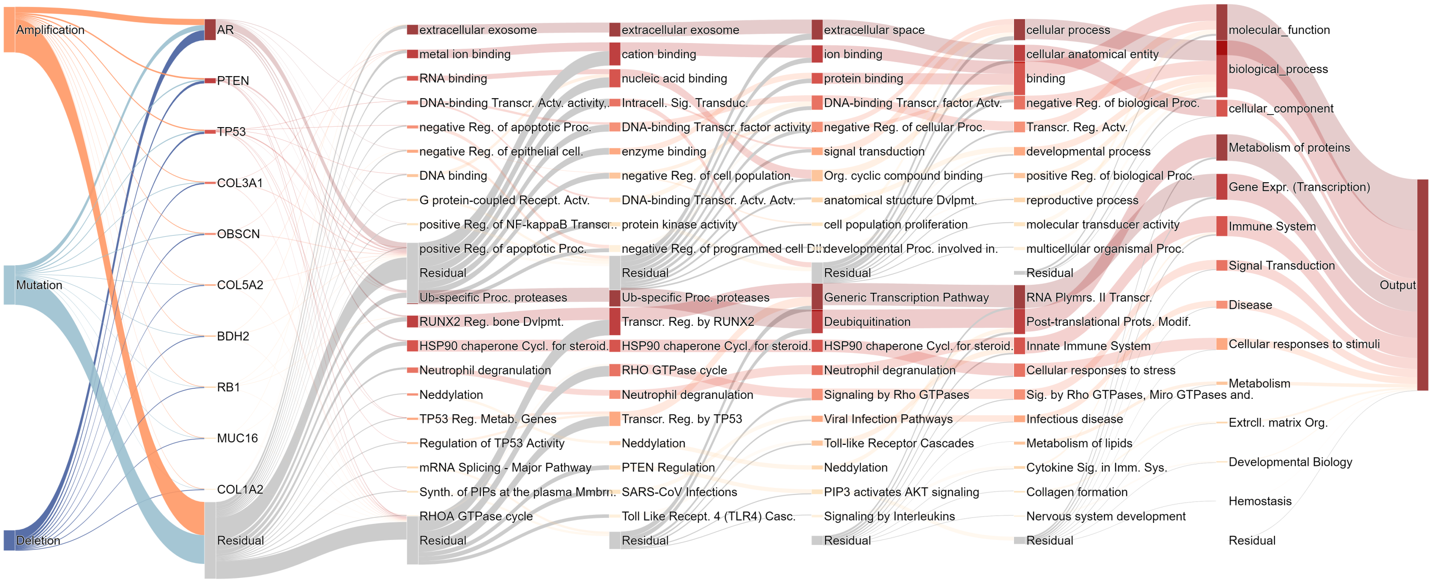

b. P-NET interpretation covers only pathways for prostate cancer.

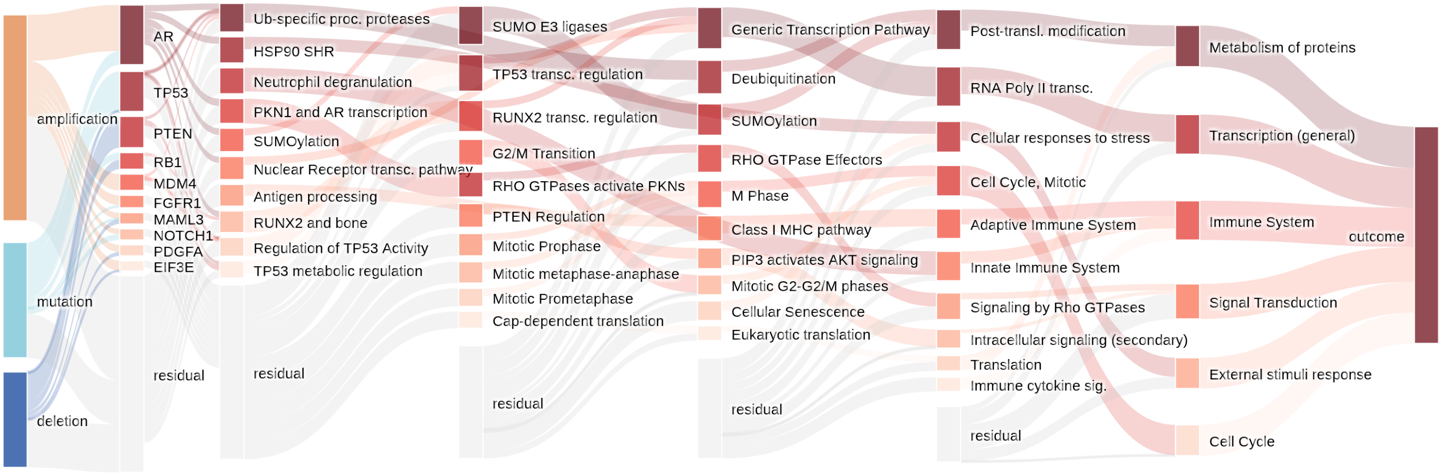

Figure S4: Comprehensive explainability analysis for prostate cancer. **a:** CLinNET and **b:** P-NET. This figure presents a comparative pathway enrichment analysis incorporating CNV deletions, amplifications, and mutations. Both models demonstrate the capacity for pathway enrichment; however, the Sankey diagrams emphasize CLinNET's broader coverage of GO terms, highlighting its enhanced ability to capture a wider spectrum of biological processes relevant to prostate cancer. Panel b was reproduced by running the publicly available source code from Elmarakeby et al.(2021)[18].

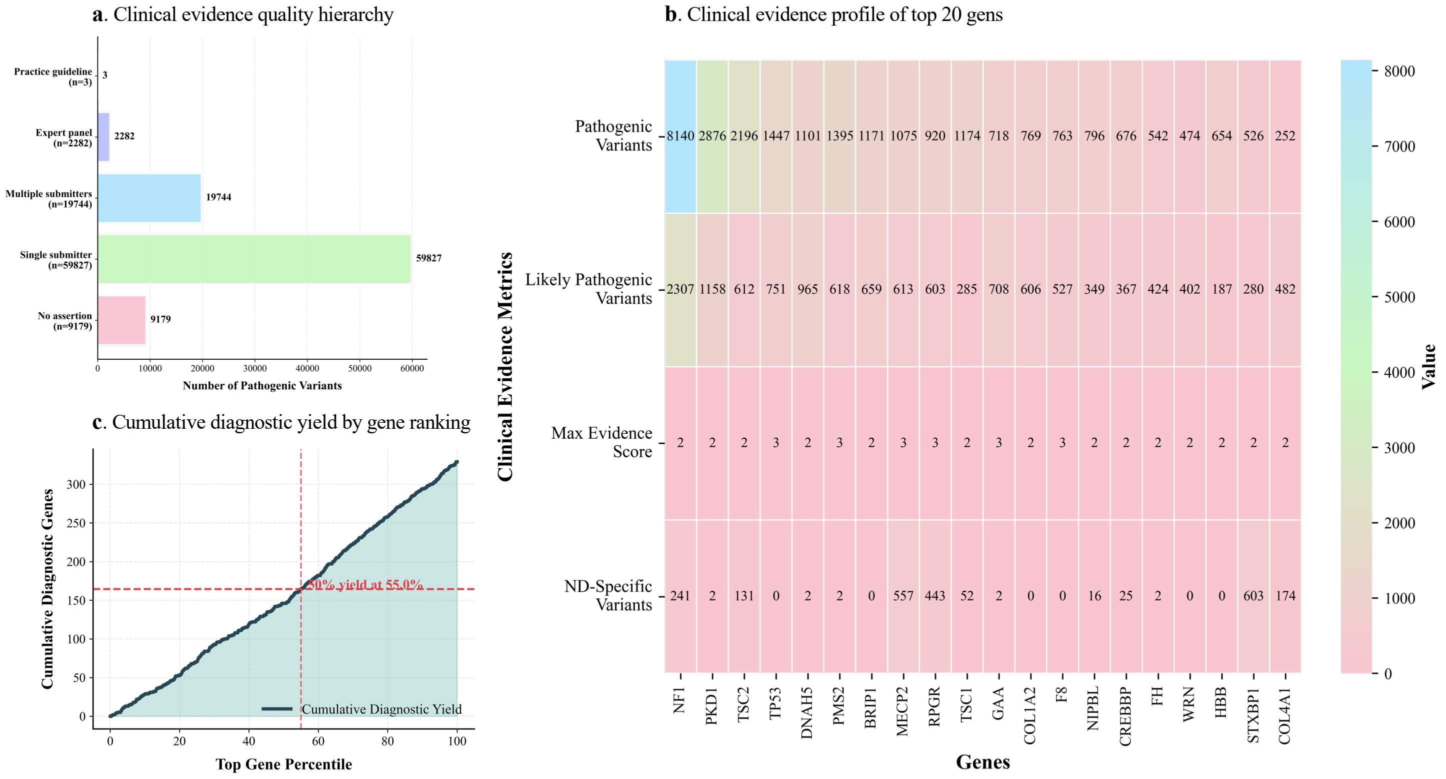

Figure S5: **a** : Diagnostic confidence levels in clinical practice. Stratification of 89,035 ClinVar-documented pathogenic cases by diagnostic confidence, illustrating the distribution of clinical evidence across hierarchical confidence tiers. Among these, 19,744 cases achieved multi-laboratory consensus, representing reproducible, cross-institutional diagnostic calls that reflect genuine clinical utility rather than site-specific variability. **b**: Clinical evidence distribution across top ClinNET-prioritized genes. Depiction of real-world diagnostic frequencies among the top 20 ClinNET-ranked genes from 89,035 pathogenic ClinVar entries. Highly ranked genes such as NF1 (8,140 cases), PKD1 (2,876), and TSC2 (2,196) exhibit concordance between model-based prioritization and real-world diagnostic burden, confirming ClinNET's ability to recover genes with established clinical impact. **c**: Clinical impact on diagnostic efficiency. Cumulative diagnostic yield curve showing that 50% of all clinically diagnosed cases can be captured by testing only 55% of ClinNet-prioritized genes (red markers). This pattern demonstrates that ClinNET's prioritization aligns with real-world diagnostic efficiency—reducing the overall gene testing burden by approximately 45%, shortening diagnostic odysseys, and improving cost-effectiveness and patient accessibility across healthcare settings.
